# Inhibition of Host Vacuolar H^+^-ATPase Activity by a *Legionella pneumophila* Effector

**DOI:** 10.1371/journal.ppat.1000822

**Published:** 2010-03-19

**Authors:** Li Xu, Xihui Shen, Andrew Bryan, Simran Banga, Michele S. Swanson, Zhao-Qing Luo

**Affiliations:** 1 Department of Biological Sciences, Purdue University, West Lafayette, Indiana, United States of America; 2 Department of Microbiology & Immunology, University of Michigan Medical School, Ann Arbor, Michigan, United States of America; Yale University School of Medicine, United States of America

## Abstract

*Legionella pneumophila* is an intracellular pathogen responsible for Legionnaires' disease. This bacterium uses the Dot/Icm type IV secretion system to inject a large number of bacterial proteins into host cells to facilitate the biogenesis of a phagosome permissive for its intracellular growth. Like many highly adapted intravacuolar pathogens, *L. pneumophila* is able to maintain a neutral pH in the lumen of its phagosome, particularly in the early phase of infection. However, in all cases, the molecular mechanisms underlying this observation remain unknown. In this report, we describe the identification and characterization of a Legionella protein termed SidK that specifically targets host v-ATPase, the multi-subunit machinery primarily responsible for organelle acidification in eukaryotic cells. Our results indicate that after being injected into infected cells by the Dot/Icm secretion system, SidK interacts with VatA, a key component of the proton pump. Such binding leads to the inhibition of ATP hydrolysis and proton translocation. When delivered into macrophages, SidK inhibits vacuole acidification and impairs the ability of the cells to digest non-pathogenic *E. coli*. We also show that a domain located in the N-terminal portion of SidK is responsible for its interactions with VatA. Furthermore, expression of *sidK* is highly induced when bacteria begin to enter new growth cycle, correlating well with the potential temporal requirement of its activity during infection. Our results indicate that direct targeting of v-ATPase by secreted proteins constitutes a virulence strategy for *L. pneumophila*, a vacuolar pathogen of macrophages and amoebae.

## Author Summary

One hallmark of the lysosome is a low luminal pH that is important for its maturation as well as the activity of many hydrolyzing enzymes responsible for efficient digestion of phagocytosed contents. To survive and replicate in phagocytes, successful intracellular pathogens have evolved various mechanisms to circumvent the challenges posed by lysosomal killing. One salient feature associated with infection of the intracellular bacterial pathogen *Legionella pneumophila* is the maintenance of a neutral pH of the Legionella containing vacuoles (LCVs) that supports its intracellular growth in the early phase of infection, while the nonpathogenic mutants are believed to be immediately trafficked to an acidic compartment. In eukaryotic cells, organelle acidification is mediated by the vacuolar H^+^-ATPase that translocates protons into target compartments in a process energized by ATP hydrolysis. The recent discovery of the association of v-ATPase with LCVs points to the necessity for active modulation of v-ATPase activity by the bacterium. By screening *L. pneumophila* proteins that cause a yeast phenotype similar to its v-ATPase mutants, we have identified a substrate of the *L. pneumophila* Dot/Icm type IV secretion system that specifically inhibits the activity of the proton transporter. This protein, termed SidK, inhibits the activity of v-ATPase by directly interacting with the VatA subunit that is responsible for hydrolyzing ATP. Moreover, macrophages harboring SidK display defects in phagosomal acidification and lysosomal killing of non-pathogenic bacteria. We also found that expression of *sidK* is highly induced right after stationary bacteria are diluted into fresh medium, suggesting that SidK plays an important role in the early phase of infection. Our results reveal a mechanism by which an intravacuolar pathogen engages the v-ATPase protein and inhibits its activity, rather than actively avoiding its association with the pathogen's vacuolar membrane.

## Introduction

The delivery of newly formed phagosomes to the lysosomal system by the endocytic pathway is essential for the digestion of phagocytosed materials. To evade such destruction, successful intracellular bacterial pathogens have evolved various mechanisms, including inhibition of phagolysosomal fusion, resistance to lysosomal digestion or the escape to the host cell cytosol. For intravacuolar pathogens, active modification of lipid and protein composition of phagosomal membrane is critical for their survival and replication. Moreover, since lysosomal enzymes often are active only in an acidic environment, regulation of pH in the phagosomal lumen is one common strategy employed by pathogens to avoid lysosomal killing [1],[2].

*Legionella pneumophila* is a facultative intracellular pathogen responsible for Legionnaires' disease. Upon being phagocytosed, this bacterium orchestrates various cellular processes to initiate a unique trafficking pathway that eventually leads to the formation of a phagosome permissive for its multiplication [3]. The biogenesis and maintenance of the bacterial replicative vacuole is mediated by protein substrates of the Dot/Icm type IV secretion system [4],[5]. For example, RalF activates and recruits the small GTPase Arf1 to the bacterial vacuole [6]. Similarly, another small GTPase Rab1 is recruited to the bacterial vacuole by SidM/DrrA, which with LepB [7], completely hijacks the activity of this important regulatory molecule in membrane trafficking [8],[9]. Whereas SidM/DrrA functions to release Rab1 from its GDI and activates the protein by loading it with GTP, LepB promotes the GTPase activity [10]. These proteins, along with other effectors such as SidJ that is involved in the recruitment of endoplasmic reticulum (ER) proteins to the bacterial vacuole [11], are thought to be responsible for the transformation of the nascent phagosome into a vacuole derived from the ER that resembles an immature autophagosome [12],[13],[14]. *L. pneumophila* also actively modulates cell death pathways of infected macrophages, presumably to ensure the well being of the host cell for a complete infection cycle. Inhibition of cell death is mediated through the activation of an NF-κB-dependent induction of antiapoptotic genes and by effectors such as SidF that directly antagonize proapoptotic BNIP3 and Bcl-rambo [15],[16],[17], and SdhA, an effector of unknown mechanism of action [18]. Effectors that modulate other cellular processes, including protein synthesis, ubiquitination and lipid metabolism have also been identified, but how the bacterium benefits from the functions of these virulence factors is less clear [19],[20],[21],[22]. Finally, a recent study indicated that the effector AnkB contributes significantly to bacterial intracellular growth but does not affect any of the above host cellular processes, suggesting the targeting of yet unidentified host pathways by *L. pneumophila* [23].

The yeast *Saccharomyces cerevisiae* has been widely used to study bacterial effectors, largely due to its genetic manipulability and the conservation of many eukaryotic cellular processes [24]. A large number of *L. pneumophila* effectors have been identified by their ability to kill yeast cells [20],[25] or to interfere with its vesicle trafficking processes [26],[27]. In eukaryotic cells, the pH of intracellular compartments is an intricately regulated parameter that is crucial for many biological processes, including membrane trafficking, protein degradation and coupled transport of small molecules [28]. Organellar acidification primarily is mediated by ATP-dependent proton transporters known as the vacuolar H^+^-ATPases or v-ATPases, which is a large multisubunit complex with an approximate molecular mass of 10^3^ kDa [28]. The structure of v-ATPases can be divided into two major functional domains: a 570-kDa peripheral subcomplex, known as V_1_, that binds and hydrolyzes ATP, and an integral membrane subcomplex, termed V_0_, that serves as the pore through which protons traverse the membrane bilayer [28]. *L. pneumophila* is able to maintain a neutral luminal pH during infection, particularly within the first 6 hrs after uptake [29],[30], a hallmark shared by many intravacuolar pathogens. One such example is *Mycobacterium avium* whose vacuoles fail to acidify below pH 6.3, probably by selectively inhibiting fusion with v-ATPase-containing vesicles or by rapidly removing the complex from its phagosomes [31]. Interestingly, a recent organelle proteomic study reveals that, in the soil amoebae host *Dictyostelium discoideum*, v-ATPase is associated with the Legionella containing vacuole (LCV) even in early phase of infection [32]. This finding is contradictory to the well-established notion that in macrophages the bacterial phagosome maintains a neutral luminal pH for several hours, suggesting that the pathogen may initially antagonize the activity of the proton transporter.

Proton transporters from different orders of eukaryotes are highly conserved in structure and function, and some genes of mammalian or plant v-ATPase components can complement the corresponding yeast mutants [33],[34]. However, whereas in mammals mutations eliminating subunits of v-ATPase generate various phenotypes, ranging from the absence of any severe phenotype to lethality to embryonic development [35],[36], yeast v-ATPase mutants are viable but only in acidic medium [37]. In this study, we have taken the advantage of this conditional phenotype of yeast *vma* mutants to identify *L. pneumophila* proteins that may target the host v-ATPases. Here we report one such protein that inhibits v-ATPase by directly interacting with one component of the proton transporter.

## Results

### Identification of SidK, a Legionella protein that affects growth of yeast cells in neutral pH medium

**Figure 1:**
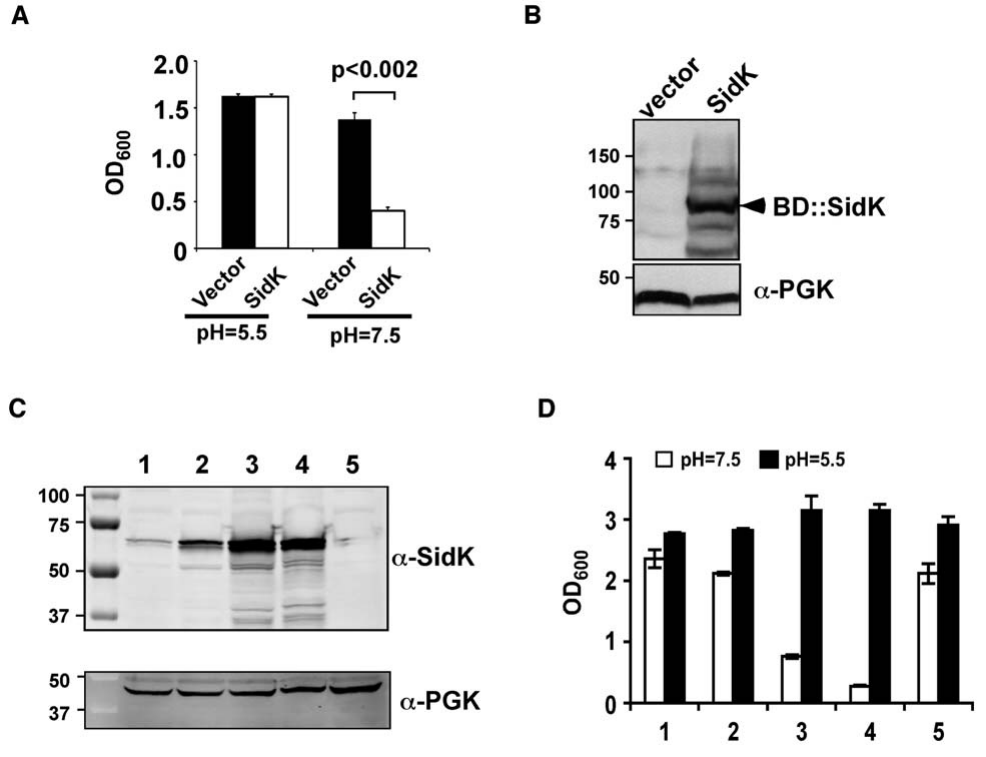
Identification of *L. pneumophila* protein that inhibits yeast growth in neutral pH medium. Yeast strains grown to saturation were diluted in medium buffered to the indicated pH at a density of 2×10^6^ cells/ml and the subcultures were incubated at 30°C with vigorous shaking. Cell growth was monitored by measuring OD_600_ 18–24 hrs after establishing the subcultures. **A.** Growth of a yeast strain expressing SidK fused to the DNA binding domain on pGBKT7. **B.** Expression of the fusion protein. Yeast strains grown to mid-log phase were lysed with a cracking buffer; SDS-PAGE resolved samples were probed with a SidK specific antibody (upper panel). The 3-phosphoglycerate kinase (PGK) was probed as a loading control (lower panel). Relevant protein size markers (in kDa) are indicated. **C.** Expression of SidK from different promoters, samples were prepared and probed as described in B (upper panel), the PGK protein was probed as a loading control (lower panel). **D.** Dose-dependent inhibition of yeast growth in neutral pH medium by SidK. The growth of yeast strains expressing SidK from vectors differing in promoter strength (Materials and Methods) was examined as described in **A**. Lanes: 1, pADH-SidK(CEN/ARS); 2, pTEF(translation elongation factor 1α)-SidK(CEN/ARS); 3, pTEF-SidK(2 μ); 4, pGPD-SidK(2 μ); 5, pGPD. Data shown are one representative experiment done in triplicates with standard variations shown.

One of the prominent phenotypes associated with yeast v-ATPase mutants is their inability to grow in neutral pH medium [37]. We reasoned that if *L. pneumophila* codes for proteins that inhibit v-ATPase activity, expression of such proteins in a yeast strain would impair its ability to grow in neutral pH medium. To this end, we cloned individual *L. pneumophila* hypothetical genes into pGBKT7 (Clontech) [20]. Yeast strains harboring each of the plasmids were tested for their ability to grow in medium with a pH of 7.5. Of the first 97 genes screened (Table S1), one gene that consistently interferes with yeast growth under this condition was obtained (Fig. 1A and B). This gene (lpg0968), designated SidK is predicted to code for a protein of approximately 65 kDa. It is present in the genomes of all sequenced strains of *L. pneumophila* but has no detectable homology to proteins in the database, nor does it contain predictable domains or motifs suggestive of known biochemical activities. Interestingly, this gene is divergently transcribed from lpg0969, a gene that appears to inhibit yeast growth by interfering with unknown host functions [27].

Since the original construct was made by fusing the testing gene to the DNA binding domain of the Gal4 protein on pGBKT7 (Clontech), we attempted to eliminate the potential effect of protein fusion by expressing untagged *sidK* in yeast strain BY4741 [38]. To this end, we used a series of vectors that differ in copy number and promoter strength (Table S3 and ref. [39]). The expression of *sidK* on these vectors is proportional to the strength of the promoter, with the GPD (glyceraldehyde-3-phosphate dehydrogenase) promoter giving the highest protein level (Fig. 1C). Consistent with the protein levels, in 18 hrs after the establishment of subcultures of identical cell density, strains in which SidK was expressed from the GPD promoter almost completely lost the ability to grow in neutral pH medium (Fig. 1D). On the other hand, only a marginal growth defect was observed when the gene was expressed from the moderate ADH promoter (Fig. 1C and D, strain 1), indicating that the effect of SidK on yeast growth under this condition is dose-dependent. Taken together, these data indicate that we have identified a *L. pneumophila* gene that affects yeast growth in neutral pH medium, probably by interfering with its v-ATPase activity directly or with activities relevant to the proton transporter.

### SidK is a substrate of the Dot/Icm system that is delivered into host cytosol during infection

**Figure 2:**
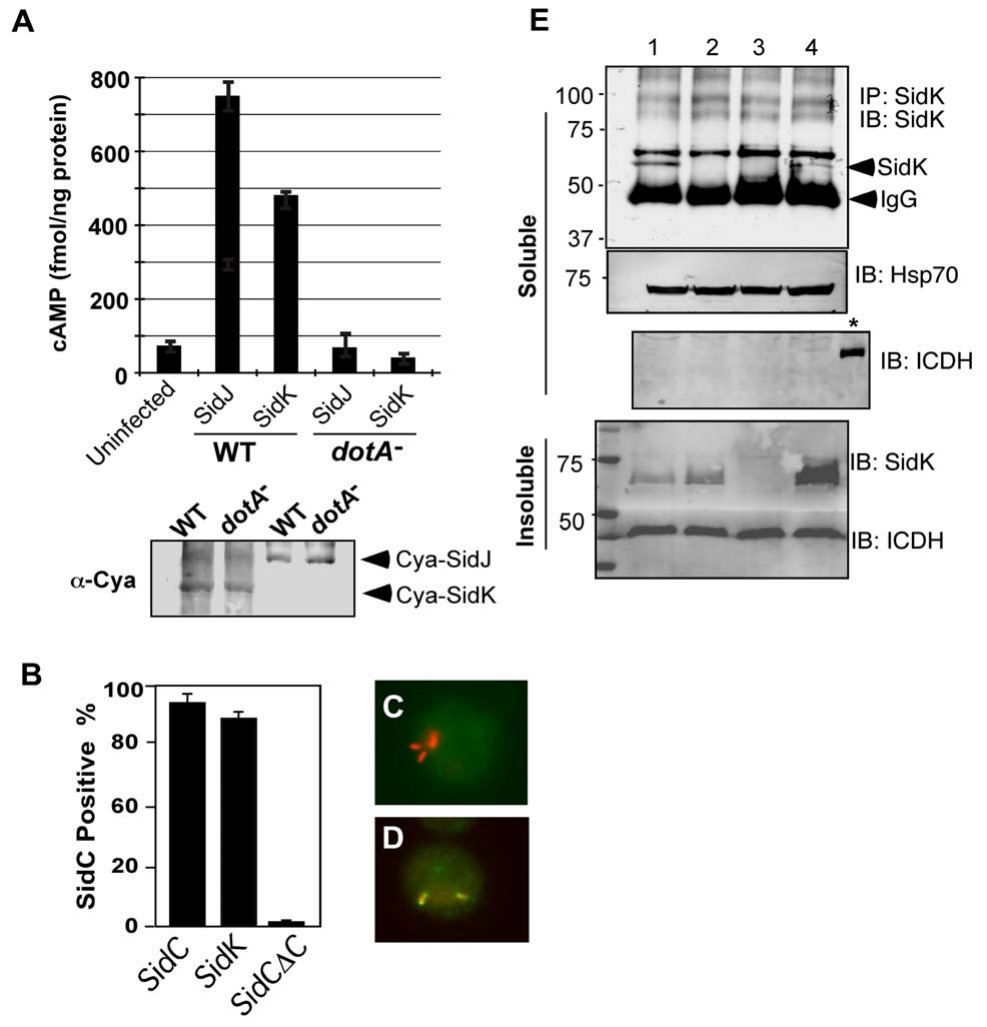
SidK is translocated into infected cells by the Dot/Icm transporter. **A.** SidK promoted the translocation of the pertusis toxin Cya into host cells. Differentiated U937 cells were infected with indicated *L. pneumophila* strains at an MOI of 5 for 1 hr. Cyclic AMP present in lysates of infected cells was measured by ELISA. Lower panel, expression of Cya fusions in *L. pneumophila* detected with a Cya specific antibody. **B–D.** Fusion to SidK restores the translocation of the transfer deficient SidΔC100. U937 macrophages were infected with bacteria expressing SidC, SidCΔC100 or SidCΔC100::SidK for one hr; *L. pneumophila* and SidC was differently labeled by immunostaining. At least 150 vacuoles in triple samples were scored in each experiment. Representative images of vacuoles harboring *sidCΔC100* (C) or *sidCΔC100::sidK*(D); bacteria were labeled in red and SidC was stained in green. **E.** Dot/Icm-dependent translocation of SidK into infected cells. U937 cells were infected with indicated *L. pneumophila* strains at an MOI of 5 for 3 hrs. Cleared supernatant obtained with 0.2% saponin was subjected to immunoprecipitation with a SidK specific antibody. Proteins associated with the precipitates were detected by the SidK antibody (upper panel). The Hsp70 in the cell lysates was probed as a loading control and the cytosolic protein ICDH was probed to assess the integrity of the bacterial cells. Note that SidK is present in pellets of all infections except for the deletion mutant (3^rd^ land, lower panel). Lanes: 1, Lp02(*dot/icm* ^+^); 2, Lp03(*dot/icm* ^−^); 3, Lp02ΔsidK; 4, Lp02ΔsidK/pSidK. *, bacterial lysate. The sizes (in kDa) of relevant protein markers are labeled on the left side of the blots.

To exert an effect on its cellular targets, a bacterial virulence factor must first reach the host cytosol via specialized secretion systems. We thus examined whether SidK is a substrate of the Dot/Icm secretion system. We first employed the Cya assay [40] by fusing SidK to the carboxyl end of the catalytic domain of the *Bordetella pertusis* cyclic AMP synthetase. Infection of macrophages with a *L. pneumophila* strain expressing Cya fused to the known effector SidJ led to production of high-level cAMP in a Dot/Icm-dependent manner (Fig. 2A). Importantly, although the Cya-SidK fusion expressed similarly in the wild type and the *dotA* mutant, high levels of cAMP were only detected in infections using the wild type strain (Fig. 2A), indicating that SidK contains signals recognizable by the Dot/Icm system. Similar results were obtained with the SidC staining assay [41], in which fusion to SidK restores the translocation of the transfer deficient SidCΔC100 mutant to wild type levels (Fig. 2B–D). These results indicate that SidK is a substrate of the Dot/Icm system.

To determine whether SidK is injected into host cells by *L. pneumophila* during infection, we attempted to detect SidK in lysates of infected cells generated by saponin fractionation [41]. Despite considerable effort, we were unable to detect this protein in the soluble fraction of lysates of cells infected by *L. pneumophila* for up to 3 hrs (data not shown). Considering the possibility that the amount of translocated SidK is beyond detection by this method; we used a SidK specific antibody to enrich the protein. After immunoprecipitation, SidK protein was detected in lysates of cells infected with wild-type strain but not with the Dot/Icm deficient mutant or a *sidK* deletion mutant (Fig. 2E, lanes 2–3). Expression of SidK from a plasmid in the *sidK* deletion mutant restored the delivery of this protein into infected cells (Fig. 2E, lane 4). Collectively, these results indicate that SidK is a substrate of the Dot/Icm system and is injected into infected cells by *L. pneumophila* during infection. Furthermore, we cannot readily detect SidK in concentrated lysates of infected cells (data not shown), suggesting that the amount of translocated protein is low.

Next, we examined the potential role of *sidK* in *L. pneumophila* infection by constructing an in-frame deletion mutant and tested its intracellular growth. The mutant did not exhibit detectable growth defect in either mouse bone marrow-derived macrophages or *D. discoideum* (Fig. S1). These observations extend the list of *L. pneumophila* effectors not essential for its intracellular growth in standard infection models, thus adding another layer to the remarkable potential functional redundancy among substrates of the Dot/Icm system [5].

Since wild type *L. pneumophila* maintains a neutral pH in its vacuole and SidK appears to interfere with the functions of v-ATPase, we analyzed whether deletion of *sidK* affects luminal pH of LCVs in mouse macrophages. Relevant *L. pneumophila* strains labeled with 5(6)-carboxyfluorescein-*N*-hydroxysuccinamide ester (FLUOS, Fluka) were used to infect macrophages and images obtained from individual phagosomes were used to calculate intravacuolar pH against a standard curve as described [30]. As expected, vacuoles containing heat-killed bacteria quickly acidified to pH values of about 4, whereas phagosomes harboring wild type *L. pneumophila* maintain a neutral pH at the time points examined (Fig. S2-A). Furthermore, although the *dotA* mutant was not lysed by the macrophages in the experimental duration (Fig. S2-B), its vacuoles were also acidified, indicating that the Dot/Icm system is required for the biogenesis of a bacterial phagosome of neutral pH. Interestingly, vacuoles containing the *sidK* deletion mutant still are able to block their acidification, thus maintaining a neutral luminal pH in the experimental duration (Fig. S2-A). Given the proficient intracellular growth displayed by the mutant (Fig. S1), this result was not unexpected. *L. pneumophila* mutants lacking a single effector gene rarely exhibit detectable intracellular growth defect, possibly due to functional redundancy among bacterial and/or host factors [5],[42].

### Expression of *sidK* is repressed at stationary phase and is induced within one hour after transition into fresh medium

**Figure 3:**
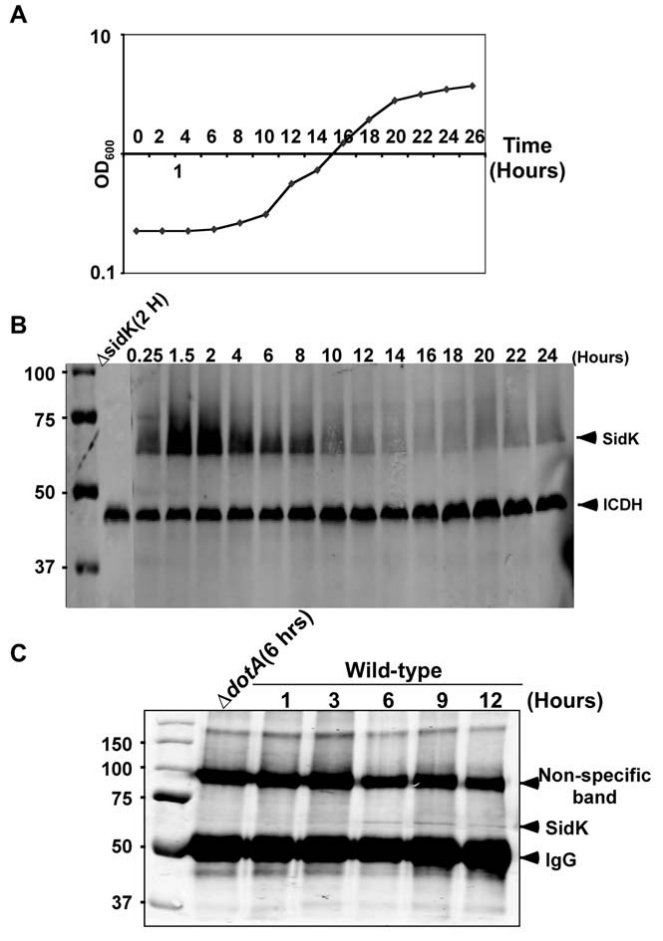
Expression of *sidK* is induced within hours at the initial phase of bacterial growth. **A.** The growth cycle of *L. pneumophila* in AYE broth. Cultures grown at stationary phase was diluted 1∶20 into fresh medium and the growth of bacteria was monitored by measuring OD_600_ at indicated time points. **B.** The expression of SidK at different bacterial growth phase. Lysates were prepared from equal amount of cells withdrawn at the indicated time points and were resolved by SDS-PAGE, the level of SidK was detected by immunoblot with a specific antibody. Lysates of the sidK deletion mutant grown for 2 hrs after dilution was used as a control. The metabolic protein isocitrate dehydrogenase (ICDH) was probed as a loading control. **C.** Kinetics of SidK translocation during infection. Lysates of U937 cells infected by the *dotA* mutant or wild type *L. pneumophila* for indicated time were immunoprecipitated with α-SidK and probed by immunoblott. Note the non-specific band about 100 kDa can serve as a loading control. The sizes (in kDa) of relevant protein markers were labeled on the left side of the blots.

Consistent with the observation that *L. pneumophila* grown at post-exponential phase are more infectious, many substrates of the Dot/Icm system are highly induced when bacterial cultures enter this growth phase [5]. That the luminal pH of LCVs is neutral in the early phase of infection points to the requirement of bacterial factors that target v-ATPase in this period of infection. We thus examined the protein level of SidK at different time points throughout the *L. pneumophila* growth cycle in broth. In contrast to many substrates of the Dot/Icm transporter whose expression is induced at post-exponential phase, very little SidK was present in *L. pneumophila* grown at this stage (Fig. 3). Rather, accumulation of SidK was apparent within 1 hr after diluting saturated cultures into fresh medium, and reached the peak 2 hrs after dilution (Fig. 3B). When the bacterium begins to replicate (approximately 4–5 hrs after dilution), protein level of SidK begins to decrease and became difficult to detect throughout the rest of the growth cycle (Fig. 3A–B), a pattern consistent with the slow progression of LCVs to lysosomal organelles [30]. We also determined the kinetics of SidK translocation during infection by saponin fractionation. Translocated SidK was not detectable until 3 hrs after infection and the protein is present in the soluble fraction of infected cells in the first 12 hrs of infection (Fig. 3C).

### SidK interacts with the host vacuolar ATPase

**Figure 4:**
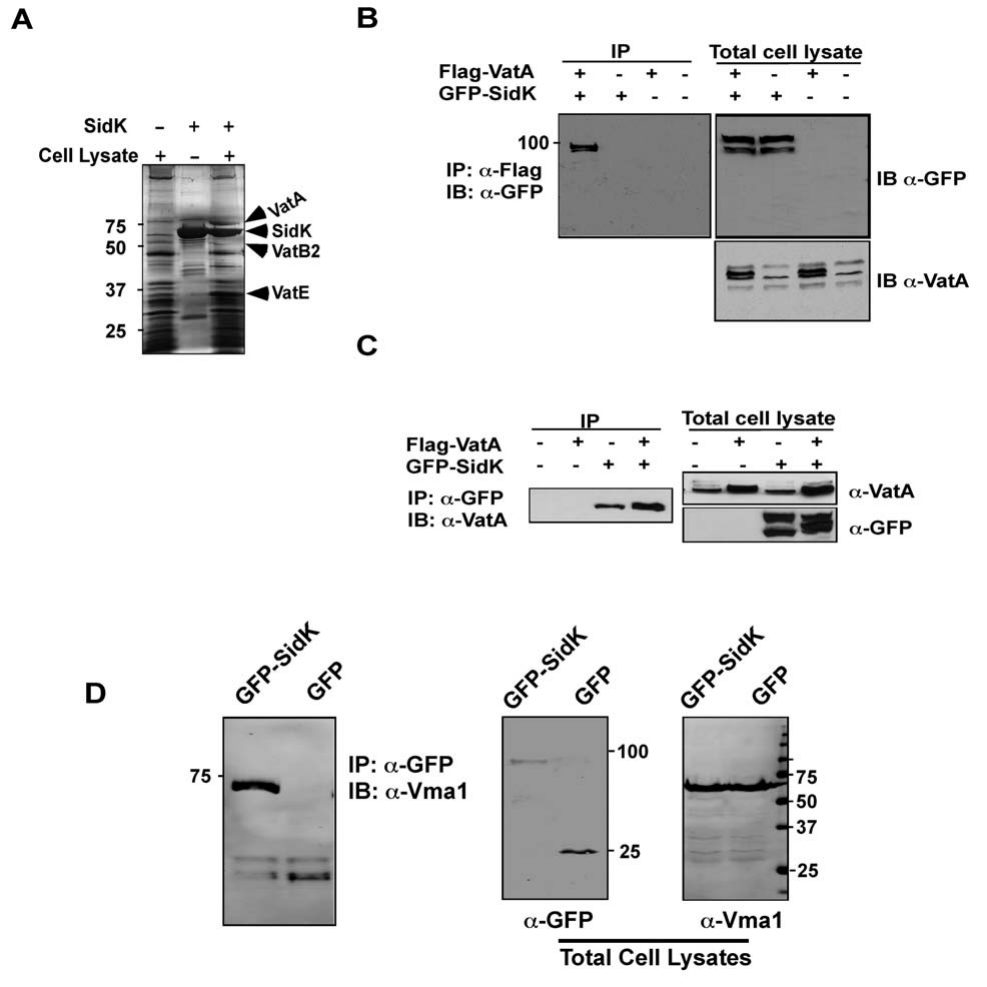
The vacuolar H^+^-ATPase is the cellular target of SidK. **A.** Subunits of the v-ATPase V_1_ domain were retained by agarose beads coated with SidK. Affigel beads blocked with Tris-HCl buffer (lane 1) or coated with SidK (lane 3) were incubated with mammalian cell lysates. SidK coated beads incubated with lysis buffer (lane 2) served as a second control. After washing with lysis buffer, proteins separated by SDS-PAGE were visualized by silver staining; bands only retained by the SidK coated beads were identified by MALDI/mass spectrometry analysis. **B.** SidK and VatA form protein complexes in mammalian cells. Lysates of 293T cells transfected to express GFP-SidK or/and Flag-VatA were subjected to immunoprecipitation with an anti-Flag antibody, the presence of SidK in precipitated proteins was detected with GFP specific antibody. **C.** SidK forms protein complexes with endogenous VatA. Lysates of cells transfected with combinations of plasmids were precipitated with a SidK specific antibody, and proteins bound to beads were detected for VatA. Note that VatA also was precipitated in cells only transfected to express GFP-SidK (lane 3). **D.** SidK formed complexes with Vma1 of yeast v-ATPase. Lysates of yeast strains expressing GFP or GFP-SidK were coimmunoprecipitated with an anti-GFP antibody, and the presence of Vma1 in the precipitates was detected. In all cases, 5% (50 µg) of total protein was probed as input controls. Relevant protein size markers (in kDa) were indicated.

Since mutations affecting various yeast genes not directly involved in v-ATPase function can result in mutants sensitive to neutral pH medium [43], we furthered our study on the mechanism of action of SidK by identifying its cellular targets with the unbiased affinity chromatograph method. We incubated Affigel beads (Bio-Rad) coated with purified SidK (Fig. S3) with PBS soluble fraction of U937 cell lysates. After removing unbound proteins by washing with PBS, proteins retained on the beads were separated by SDS-PAGE and were visualized by silver staining. At least three proteins with molecular weights ranging between 20 kDa and 75 kDa were retained by beads coated with SidK, but not by uncoated beads (Fig. 4A, lane 3). By mass spectrometry analysis, we identified these proteins as three components of the mammalian vacuolar H^+^-ATPase: VatA, the ubiquitous VatB_2_ subunit and VatE (Fig. 4A, lane 3). These results indicate v-ATPase is the potential target of SidK.

To further investigate the interactions between v-ATPase and SidK, we transfected mammalian cells with combinations of plasmids that direct the expression of GFP-SidK or Flag-VatA, one essential component of the proton transporter [28]. Lysates of transfected cells were subjected to co-immunoprecipitation (co-IP) with an anti-Flag antibody to detect possible SidK/VatA complexes. GFP-SidK was detected in immunoprecipitates only from cells coexpressing Flag-VatA (Fig. 4B). No signals were detected in untransfected samples or in samples transfected with plasmids expressing only Flag-VatA or GFP-SidK, indicating that the interactions were specific. Similar results were obtained in reciprocal experiments using the anti-GFP antibody (Fig. 4C). Furthermore, VatA was detected in precipitates from cells that was transfected only with the plasmid expressing SidK, indicating that this protein interacts with endogenous VatA (Fig. 4C, lane 3).

The structure and function of v-ATPase from mammals and yeast are highly similar [28]. We thus examined whether SidK interacts with yeast v-ATPase. When lysates of yeast cells expressing GFP or GFP-SidK were immunoprecipitated with a GFP-specific antibody, Vma1 (equivalent of VatA) was detected in precipitates, again only in samples expressing SidK (Fig. 4D, lane 1 in left panel). Similar results were obtained in reciprocal immumoprecipitation using a Vma1 specific antibody (data not shown). Taken together, these data establish that v-ATPase is the cellular target of SidK.

### SidK directly interacts with the VatA (Vma1) subunit of the v-ATPase

**Figure 5:**
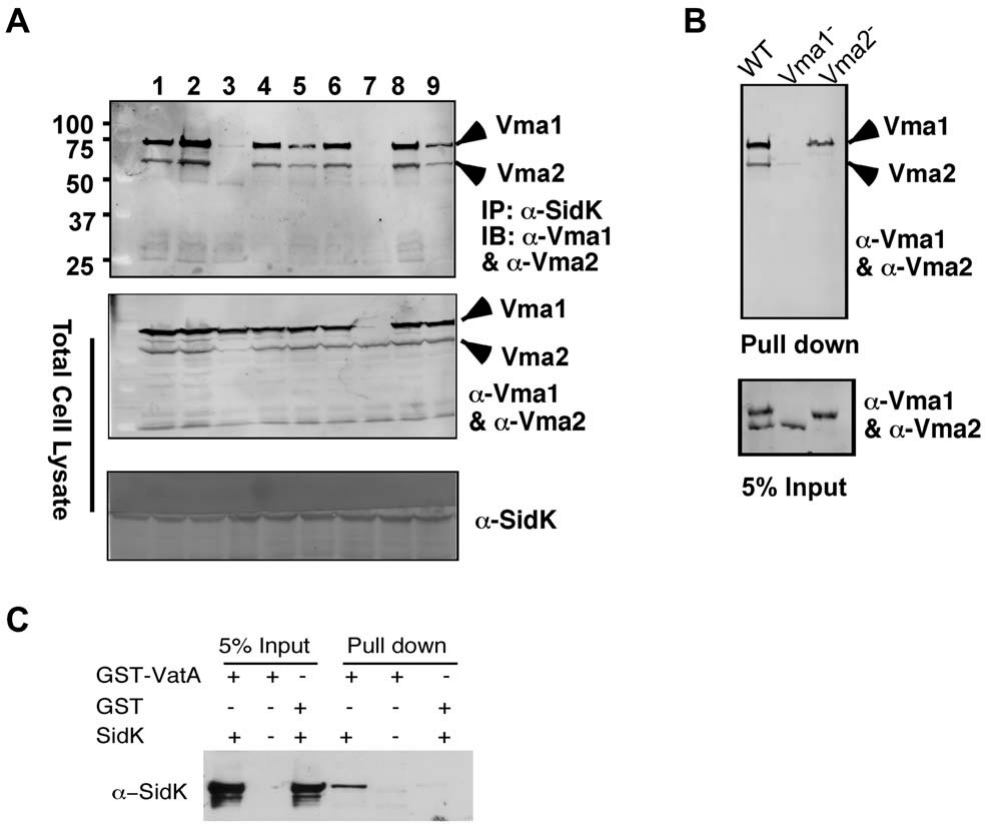
SidK directly interacts with VatA, the ATP hydrolyzing subunit of v-ATPase. **A.** SidK interacts with Vma1 in the absence of other V_1_ subunits. Lysates of individual *vma* mutant expressing SidK were coimmunoprecipitated with the anti-SidK antibody and the presence of Vma1 and Vma2 in the precipitates were detected with specific antibodies. Lanes: 1, *Δvma4*; 2, wild type; 3, Δ*vma2*; 4, Δ*vma5*; 5, Δ*vma7*; 6, Δ*vma8*; 7, Δ*vma1*; 8, Δ*vma10*; 9, Δ*vma13*. The presence of SidK, Vma1 and Vma2 in the samples were probed with 5% proteins used for immunoprecipitation. **B.** SidK coated beads retained Vma1. Affigel beads coated with SidK were incubated with cell lysates of wild type *Δvma1* or *Δvma2* mutant. Proteins associated with the beads after extensive wash were probed for Vma1 and Vma2. 5% of lysates was used to probe for the presence of these proteins. Relevant protein size markers (in kDa) were indicated. **C.** SidK directly interacts with VatA. His_6_-SidK was incubated with GST-VatA or GST in PBS and potential protein complexes captured with glutathione beads were detected with specific antibody.

Under normal physiological condition, components of the V_1_ domain of v-ATPases form a stable complex [28]. In agreement with this notion, in immunoprecipitation experiments aiming at detecting interactions between SidK and components of the V_1_ complex, positive interactions were observed in many if not all components (data not shown). Thus, we further investigated which subunit of the V_1_ domain is directly targeted by SidK. Because some V_1_ components are recalcitrant to purification in their soluble form, we used yeast mutants that lack individual V_1_ component genes [38] to identify the subunit that directly interacts with SidK. We first examined the formation of protein complexes between SidK and two V_1_ subunits, Vma1 and Vma2 in these mutants. Vma1 and Vma2 can be coimmunoprecipitated by the SidK antibody in mutants lacking *vma*4, 5, 7, 8, 10 or 13, indicating that none of these subunits is required for the formation of protein complexes between SidK and Vma1 or Vma2 (Fig. 5A). However, in the absence of Vma1, no interactions between SidK and Vma2 or any other V_1_ components were detected (Fig. 5A, lane 7 and data not shown). Importantly, although at a low level, Vma1 was detected in precipitates obtained by the SidK antibody in the *vma2* mutant (Fig. 5A, lane 3). Furthermore, when beads coated with SidK were incubated with lysates of different *vma* mutants, Vma1 from the lysates of the *vam2* mutant was retained (Fig. 5B, lane 3). Under the same condition, SidK coated beads did not retain Vma2 or other V_1_ components expressed in the *vma1* mutant (Fig. 5B, lane 2 and data not shown). Collectively, these results point to Vma1 as the direct target of SidK. To confirm this conclusion, we purified recombinant mammalian VatA as a GST tagged protein (GST-VatA) and tested its interaction with SidK. As expected, formation of SidK/GST-VatA complexes can be captured by GST beads (Fig. 5C). From these results, we conclude that SidK targets the v-ATPase by directly interacting with the VatA (Vma1) subunit.

### A N-terminal domain of SidK is important for binding to Vma1

**Figure 6:**
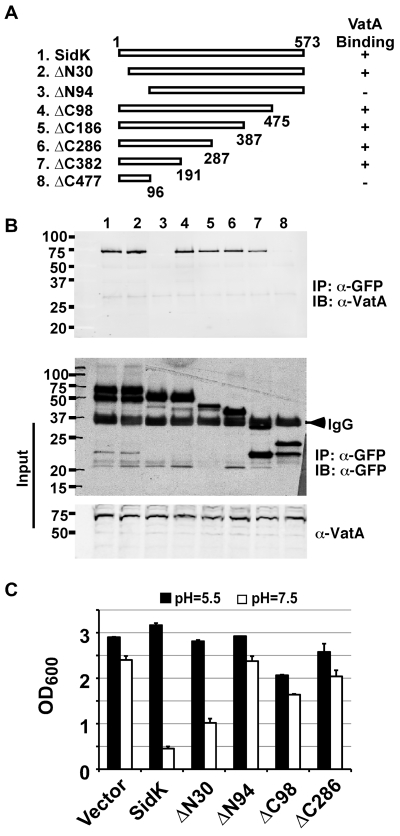
SidK binds to VatA via a N-terminal domain. **A.** Diagrams of *sidK* truncation mutants. The numbers at the ends of the bars are the numbers of remaining amino acids for the mutants. +: binds VatA; -, no longer binds VatA. **B.** Interactions between VatA and the SidK deletion mutants. Lysates of 293T cells expressing each of the mutants fused to GFP were subjected to coimmunoprecipitation with an anti-GFP antibody and the presence of VatA in the precipitates were probed. The middle panel shows protein levels of the mutants probed with a GFP specific antibody after immunoprecipitation. Note that the several mutants that no longer interact with Vma1 still code for stable proteins. Endogenous VatA also was probed as input controls (lower panel). **C.** Inhibition of yeast growth in neutral pH medium by SidK mutants. Indicated mutants (without any tag) were cloned into p425GPD and yeast growth was assayed as described in Fig. 1.

We extended our analysis of the interactions between SidK and VatA by determining the region on SidK important for target binding. To this end, we constructed a series of SidK deletion mutants (Fig. 6A). To eliminate the potential discrepancy that may arise from the loss of epitopes in these mutants when the polyclonal anti-SidK antibody is used in subsequent experiments, we expressed GFP fusions of these mutants in mammalian cells. Deletion of 30 amino acids from the N-terminus of SidK did not detectably affect its binding with VatA (Fig. 6B, lane 2). However, although it was expressed at a high level, a mutant missing the first 94 amino acids no longer detectably bound VatA (Fig. 6B, lane 3). On the other hand, the VatA binding activity of SidK is far more tolerant to deletions in its carboxyl end. A mutant lacking as many as 382 amino acids from this end of SidK still co-precipitated with VatA at a level similar to that of the full-length protein (Fig. 6B, lane 8). Similar results were obtained in yeast, with the exception of sidKΔC382, which did not bind Vma1, probably due to the different binding affinity of SidK to v-ATPases from these two organisms (Fig. S4). Collectively, these results indicate that a domain that lies within residue 30 to 191 of SidK is important for interacting with VatA.

To determine whether any of the SidK deletion mutants are still active in inhibiting v-ATPase functions, we tested their ability to inhibit yeast growth in neutral pH medium. Our results indicate that under this condition, SidKΔN30 consistently inhibits yeast growth, whereas the binding inactive mutant SidKΔN94 exhibits very little effect (Fig. 6C). Similarly, the binding of SidKΔC98 and SidKΔ286 to VatA is comparable to that of full-length protein and both mutants exhibit detectable inhibition in yeast growth (Fig. 6C). Although these mutants can be stably expressed in yeast from the GPD promoter, their expression levels are considerably lower than that of full-length SidK (Fig. S5). Since high protein level is required for SidK to exert full inhibitory effect on yeast growth (Fig. 1), the low activity exhibited by these mutants may result from the their low protein levels in yeast.

### SidK inhibits v-ATPase-mediated ATP hydrolysis and proton translocation

**Figure 7:**
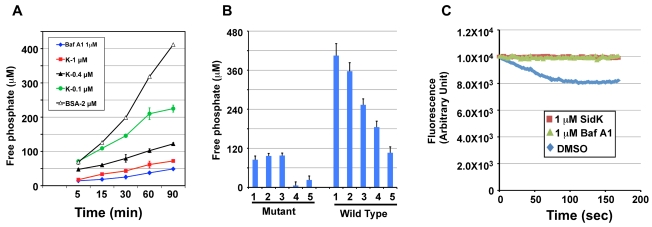
SidK inhibits v-ATPase-mediated ATP hydrolysis and proton translocation. **A.** SidK inhibits ATP hydrolysis in yeast vesicle membranes. BSA, Baf A1 or three different concentrations of His_6_-SidK was incubated with yeast vesicle membranes, ATP was added to initiate the reaction. The concentration of free phosphate in samples withdrawn at indicated time points was determined by the malachite green method (Materials and Methods). **B.** SidK specifically inhibits ATPase activity of v-ATPase. Yeast vesicle membranes prepared from wild type or the *vma1* mutant were used for similar assays. Note that SidK and the v-ATPase specific inhibitor Baf A1 block ATPase activity in membranes from the wild type but not the *vma1* mutant. Lanes (mutant): 1, 1 µM SidK; 2, 1 µM Baf A1; 3, 1 µM BSA; 4, 10 mM EDTA; 5, 1 mM vanadate. Lanes (wild type): 1, 1 µM BSA; 2, 0.1 µM SidK; 3, 0.4 µM SidK; 4, 1 µM SidK; 5, 1 µM Baf A1. **C.** SidK prevents v-ATPase-mediated proton translocation. ATP was added to reactions containing vacuolar membrane vesicles, acridine orange and the indicated reagents that had been preincubated for 40 min at room temperature. The quenching of acridine orange fluorescence was monitored as described in Experimental Procedures.

The V_1_ domain of v-ATPase provides the energy required for proton translocation across membranes by binding and hydrolyzing ATP via subunit Vma2 and Vma1, respectively [44]. Our observation that SidK directly binds Vma1 prompted us to examine the effect of such binding on ATP hydrolysis. Thus, we followed a standard procedure [45] to prepare yeast membrane and examined the effect of SidK on its ATPase activity. In this assay, exogenous ATP was added to purified yeast membranes and the release of free phosphate was measured by malachite green [46]. In samples receiving 2 µM BSA, the level of free phosphate steadily increased throughout the experimental duration (Fig. 7A, open triangles). On the other hand, the addition of 1 µM bafilomycin A1 (Baf A1), a commonly used inhibitor of v-ATPase [47] led to strong inhibition of ATP hydrolysis, thus low level of free phosphate (Fig. 7A, diamonds). Importantly, we found that recombinant SidK inhibited v-ATPase activity in a dose-dependent manner. Significant inhibition was observed by 0.1 µM SidK, and a higher amount of protein caused more severe inhibition (Fig. 7A, closed triangles); 1 µM of SidK exerted inhibition at a level similar to that of Baf A1 (Fig. 7A, squares). Next, we probed the mechanism of action of SidK by adding its antibody to the reactions. The antibody did not detectably affect SidK activity even when it was two-fold in excess (Fig. S6).

To confirm that the observed inhibition of ATP hydrolysis was indeed a result of blocking v-ATPase activity, we did similar experiments with membranes prepared from the *vma1* mutant. For the same amount of membranes, the overall ATP hydrolysis activity of the mutant markedly decreased (compare 3^rd^ bar of mutant and 1^st^ bar of WT in Fig. 7B). Moreover, the ATP hydrolysis activity of membranes from the mutant is resistant to Baf A1 or SidK, but not to EDTA and vanadate, two general inhibitors for ATPases [48] (Fig. 7B, mutant 4^th^ & 5^th^ bar). On the other hand, ATPase activity in membranes prepared from wild type yeast consistently exhibited sensitivity to SidK, again in a dose-dependent manner (Fig. 7B, wild type 2^nd^-5^th^ bar). We also tested the effect of SidK on the ATP hydrolysis activity of mammalian Hsp70, an ATPase structurally distant from the v-ATPase [49]. EDTA but not SidK abolished ATP hydrolysis by this heat shock protein; vanadate also exhibited inhibitory effect, but to a lesser extent (Fig. S7). Taken together, these results indicate that binding of SidK to Vma1 leads to specific inhibition of the ATP hydrolysis activity of the proton transporter.

Because v-ATPase-mediated ATP hydrolysis is coupled with proton translocation, and thus the acidification of vesicles [50], inhibition of v-ATPase activity by SidK would block vesicle acidification. To test this hypothesis, we determined the effect of SidK on the sequestration of the lipophilic amine acridine orange (AO) by yeast vesicles. Nonprotonated AO permeates membranes, and, if the pH of the vesicles drops as a result of v-ATPase-mediated proton translocation, it becomes protonated and sequestered, leading to quenching of its fluorescence [51]. In samples receiving the solvent DMSO that does not affect v-ATPase activity, more AO was trapped in the vesicles as proton translocation was initiated by adding ATP, leading to quenching of fluorescence at 525 nm (Fig. 7C, diamonds). On the other hand, inclusion of 1 µM SidK to the reaction blocked such quenching during the entire experimental duration (Fig. 7C, squares). The effect of SidK at this concentration is comparable to that of Baf A1, which almost completely blocked AO fluorescence quenching (Fig. 7C, triangles). From these observations, we conclude that inhibition of ATP hydrolysis activity of v-ATPase by SidK prevents proton translocation.

### SidK interferes with phagosome acidification and phagolysosomal digestion of bacteria

**Figure 8:**
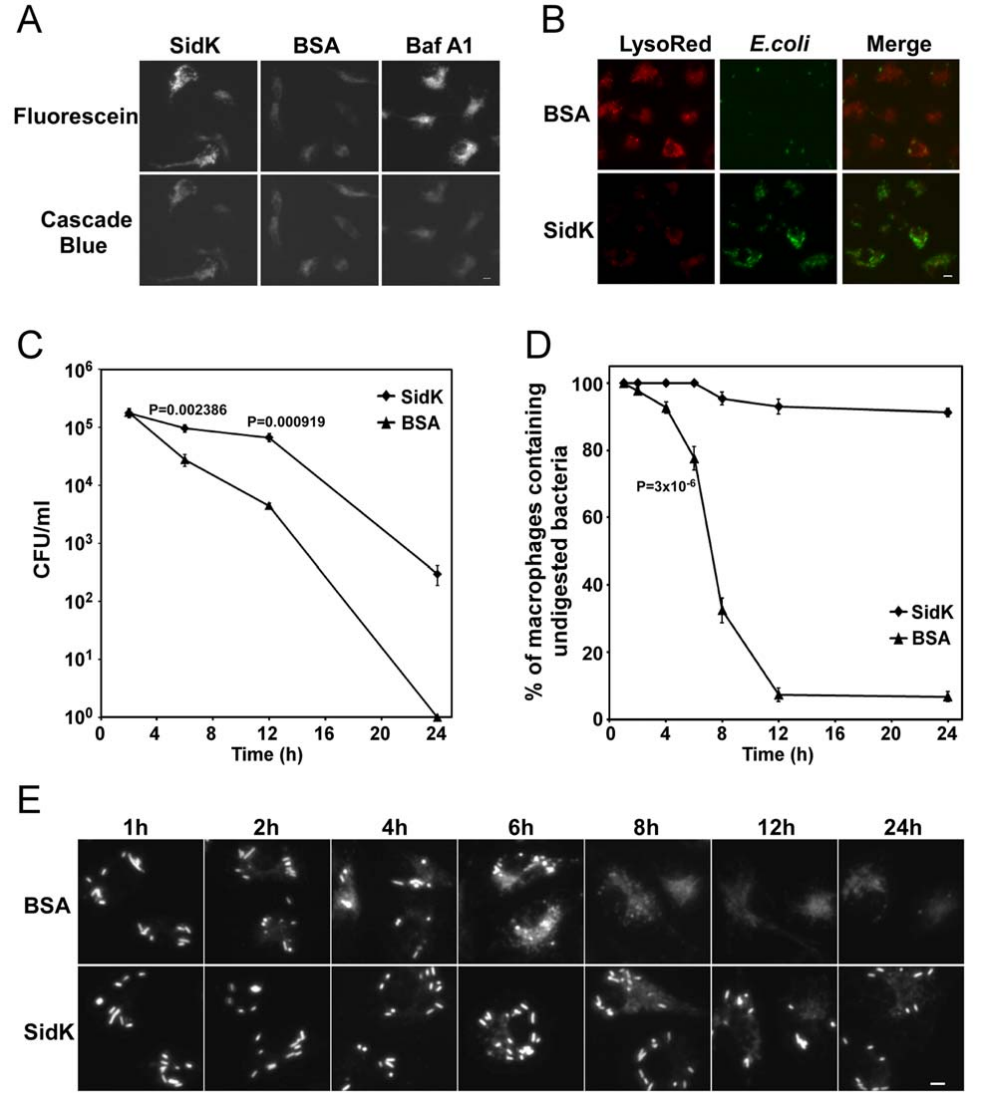
Macrophages loaded with SidK are defective in phagosomal acidification and lysosomal digestion of bacteria. **A–B.** Phagosomal acidification in macrophages assessed by pH sensitive fluorescein dextran and LysoRed staining. Mouse bone marrow-derived macrophages were loaded with His_6_-SidK or BSA by syringe loading. Treated cells were incubated with a mixture of fluorescein dextran and cascade blue dextran (0.2 mg/ml) for 1 h, washed 5 times and incubated at 37°C for 4 hrs before being imaged. As a control, Baf A1 (250 nM) was added 40 min before taking the images (A). *E. coli* cells expressing GFP were incubated with loaded macrophages at an MOI of 20 for 1 hr at 37°C. After incubating with gentamicin for 8 hrs, cells were stained with LysoRed (50 nM) for 15 min. Images were acquired using a fluorescence microscope with identical parameters (B). Bar, 5 µm. **C.** SidK affects bacterial killing by macrophages. Loaded macrophages were fed with *E. coli* cells expressing mCherry RFP at an MOI of 10 for 1 hr at 37°C. Samples were treated with gentamicin for 1 h and were extensively washed. Viable *E. coli* cells were evaluated by plating macrophage lysates on bacteriological media at indicated time points. **D.** Digestion of bacteria by macrophages. Samples prepared similarly as C were used to evaluate the ratios of macrophages that contain intact *E. coli* cells. Cells harboring one or more bacterial cells were quantitated at indicated time points. Experiments were performed in triplicates and at least 200 cells were examined each sample. The *P* values of the relevant data points are indicated. Similar results were obtained in more than three independent experiments. **E.** Representative images of macrophages fed with fluoresent *E. coli* cells. Samples at indicated time points were processed and analyzed under a fluorescence microscope and images of typical cells were acquired. Bar, 5 µm.

To determine whether SidK affects the functions of v-ATPase *in vivo*, we delivered His_6_-SidK into mouse bone marrow-derived macrophages by syringe loading [52] and examined the acidification of phagosomes by using the dextran-coupled pH sensitive fluorescein, whose fluorescence drops very sharply at pH values below 5.5 [53]. The pH insensitive Cascade Blue dextran was included in the feeding mixture as a loading control. Macrophages in all samples emitted blue fluorescence signals at similar intensity, indicating that the dyes were equally loaded into the cells (Fig. 8A, lower panel). Importantly, compared to macrophages receiving BSA, cells loaded with SidK gave significantly stronger green fluorescence signals (Fig. 8A, upper panel, the first two images). Similarly, compared to cells loaded with BSA, cells treated with the v-ATPase inhibitor Baf A1 also emitted stronger fluorescence signals (Fig. 8A, upper panel, the right image). These results indicate that SidK interferes with efficient acidification of phagosomes, thus inhibiting the decrease of their luminal pH values. To substantiate this observation, we used LysoRed, which accumulates in acidified organelles to stain protein-loaded macrophages that have been fed with *E. coli* cells expressing GFP for 10 hrs. Strong red fluorescence signals were readily detected in cells loaded with BSA, but not in cells receiving SidK (Fig. 8B, left panel), further supporting the notion that SidK inhibits phagosomal acidification. No effect was detected in additional controls with two other Legionella effector proteins (data not shown). Moreover, at this time point, we observed that the number of *E. coli* cells in macrophages loaded with SidK was significantly higher than that of cells containing BSA (Fig. 8B, middle panel), suggesting that inhibition of phagosomal acidification by SidK impaired the lysosomal digestion of internalized bacteria.

Since macrophages from mice lacking a functional a3 subunit exhibit delayed digestion of bacteria [54], we set to more thoroughly examine the effect of SidK on macrophage-mediated lysis of *E. coli* cells. We first determined the survival of *E. coli* in macrophages loaded with different proteins. Macrophages loaded with His_6_-SidK or BSA are capable of killing phagocytosed bacteria, but cells containing SidK were less efficient in the clearance of the bacteria, and such differences became significant 6 hrs after adding the bacteria (Fig. 8C). Similar results were obtained when macrophages harboring one or more intact *E. coli* cells were scored. In cells receiving His_6_-SidK, more than 90% of the cells harbor intact bacterial cells throughout the 24 hrs experiment duration (Fig. 8D). However, 8 hrs after adding the bacteria, less than 40% of the macrophages loaded with BSA contained intact bacterial cells and the ratio of such cell population dropped to less than 10% at the12-hr time point (Fig. 8D and E). Taken together, these results indicate that SidK can inhibit v-ATPase activity *in vivo* and such inhibition leads to defects in phagosomal acidification and impairment in lysosomal digestion of bacteria by macrophages.

## Discussion

Many intracellular bacterial pathogens reside and replicate in phagosomes of unique physiological and biochemical properties. One such property is an actively regulated pH homeostasis important for successful infection of these pathogens. Since the vacuolar ATPase is the primary cellular machinery involved in controlling vacuolar pH [50], it is believed that pathogens capable of maintaining a neutral phagosomal pH encode specific traits to inhibit the accumulation of the proton transporters on their vacuolar membranes. However, although the importance of maintaining proper phagosomal pH, presumably by actively regulating the activity of v-ATPase, is generally recognized, almost nothing is known concerning the molecular mechanisms responsible for such regulation. Using a screening strategy based on the sensitivity of yeast v-ATPase mutants to neutral pH medium, we have identified SidK, a *L. pneumophila* protein that targets the proton transporter.

A pathogen can employ at least two mechanisms to maintain a neutral luminal pH in its phagosomes: By preventing the accumulation of v-ATPases on the phagosomal membranes or by inhibiting the activity of acquired v-ATPases. Although we have not been able to consistently detect SidK on LCVs, probably because of low protein level and/or poor antibody quality (Fig. 2 and data not shown), the presence of v-ATPases on LCVs [32] strongly suggests that SidK targets the proton pumps on the bacterial phagosomes. This feature differs from vacuoles of *Mycobacterium ovium* that do not contain detectable v-ATPases [31]. However, these two mechanisms are not mutually exclusive, because in addition to blocking its acquisition, the pathogen may need to antagonize v-ATPases that accidentally associate with its phagosomes. It is worth noting that detecting the association of v-ATPase with specific organelles can be complicated by low abundance of this protein complex on the membranes. For example, only a few v-ATPases were detected on a phagosome containing a latex bead [55]. Similarly, v-ATPases associated with LCVs can be detected by the sensitive mass spectrometry but not by standard immunostaining (ref. [32] and data not shown). Thus, direct targeting of v-ATPase by specific virulence factors could be a mechanism shared by many intravacuolar pathogens.

Our data showed that SidK interacts with v-ATPases by directly binding to the VatA subunit (Fig. 5). Moreover, this protein appears to have a higher affinity for VatA in the presence of VatB (Fig. 5A). Such differences may result from the conformation assumed by VatA when it is associated with VatB [28]. Preferably binding to the VatA/VatB and/or the fully assembled v-ATPase complex clearly will result in higher inhibitory efficiency for SidK, as its effect can be diluted by free VatA if these two proteins interact similarly regardless of their statuses. Reversible assembly of the V_1_ and V_0_ domain is important in regulating v-ATPase activity under different physiological conditions [28]. Whether binding of SidK to v-ATPase causes disassembly of the transporter, block of its rotary movement or other functional mechanisms of the proton transporter remains to be determined. Our biochemical studies indicate that SidK blocks organelle acidification during infection. Two lines of evidence indicate that SidK inhibits v-ATPase *in vivo*. First, macrophages harboring physically delivered recombinant SidK failed to block emission of fluorescence signals by the pH sensitive fluorescein (Fig. 8A). Second, cells loaded with SidK sequester significantly lower levels of LysoRed, a fluorescence dye that prefers to accumulate in acidic environments (Fig. 8B). Moreover, similar to macrophages from mice lacking a subunit of the v-ATPase [54], cells loaded with SidK displayed a significant delay in the digestion of phagocytosed bacteria (Fig. 8C–E). Thus, it is clear that by binding to VatA, SidK is able to block phagosomal acidification, thus contributing to the protection of internalized *L. pneumophila* during infection.

Bacterial effectors often enzymatically modify their targets to divert the cellular processes in ways beneficial to the survival of the pathogens [42]. However, despite considerable effort, we were unable to detect novel post-translational modifications on VatA co-purified with SidK from yeast (data not shown). Moreover, our attempt to determine the mechanism of action of SidK by its antibody is not conclusive because the antibody is able to immunoprecipitate Vma1 or VatA, indicating that it can form a stable complex with these two proteins (Fig. 5A and data not shown). Although SidK-mediated modifications of VatA or other v-ATPase subunits could substantiate its effect, two lines of evidence indicate the importance of physical binding in the activity of SidK. First, in contrast to other highly effective *L. pneumophila* effectors, such as those involved in inhibiting host protein synthesis or membrane trafficking [5], a much higher level of SidK is required to significantly inhibit yeast growth in neutral pH medium, a condition that is completely unable to support growth of yeast *vma* mutants (Fig. 1). For instance, if the effect of SidK was mediated by a highly catalytic mechanism, one would expect more severe inhibition when expressed from the ADH (alcohol dehydrogenase) promoter (Fig. 1C). Similarly, a considerable amount of SidK is needed to inhibit v-ATPase activity in yeast membranes (Fig. 7). Second, some deletion mutants capable of binding VatA still are able to exert inhibitory effect on yeast growth in neutral pH medium (Fig. 6C). Given the requirement of high-level SidK for full growth inhibition, the loss of inhibitory effect by deletion mutants still competent for binding VatA very likely is a result of lower protein levels (Fig. S5). Alternatively, SidK may need other *L. pneumophila* proteins to exert its full activity or our experimental conditions are not optimal for its activity.

In A/J mouse macrophages, vacuoles containing *L. pneumophila* become acidified during the replication phase of infection. Delayed maturation of the LCV promotes intracellular growth, since Baf A1 treatment blocks acidification and acquisition of lysosomal markers and also reduces the bacterial yield [30]. Consistent with the observation that expression of *sidK* peaks early in the lag phase before declining to almost undetectable levels as the bacteria enter the post-exponential phase, translocated SidK did not become detectable until 3 hrs after bacterial uptake. That translocated SidK is still detectable 12 hrs after infection suggests a delayed inhibition of SidK expression during infection or that SidK expresses differently in intracellular bacteria. In *D. discoideum*, the association of v-ATPases with the LCV is detectable from 15 min to 14 hrs after bacterial uptake [32]. The presence of this transporter in the early phase of infection suggests that the undetected SidK and/or other effectors antagonize its activity. Given the important roles of v-ATPase in vesicular trafficking, particularly in the endocytic pathway [56] that was recently shown to be involved in remodeling the membranes of the LCVs in *D. discoideum* [32], prolonged biochemical modifications of the v-ATPase may interfere with the ability of the bacteria within phagosomes to efficiently acquire nutrients and materials from certain membrane trafficking pathways. Thus, *L. pneumophila* appears to use a combination of strategies to modulate the activities of v-ATPase at different phases of infection for its benefit.

*L. pneumophila* appears to acquire many of its genes important for its interactions with host by horizontal gene transfer, which may account for at least in part the high plasticity of its genomes [57],[58]. Although the distinct biochemical activities of these genes can interfere with host cellular processes, a single gene often plays only a small incremental role in its evolution to parasitize its hosts, which may explain the remarkable functional redundancy among effectors of the Dot/Icm system [5],[42]. For example, at least four proteins are involved in inhibiting host protein synthesis by targeting the elongation factor eEF1A [20],[59]. Consistent with this notion, with a few exceptions, deletion of one or more Dot/Icm substrate genes did not cause detectable defect in intracellular growth [5],[42],[60]. Thus, our observation that deletion of *sidK* did not impair intracellular growth of *L. pneumophila* or its phagosomal pH is not completely unexpected. It is very likely that multiple Dot/Icm substrates are involved in the modulation of v-ATPase activity. Identification and elucidation of activities of such proteins should pave the way toward further understanding of the mechanisms underlying organelle acidification and of the means whereby it can be disrupted by pathogens.

## Materials and Methods

All animal use procedures were in strict accordance with the NIH Guide for the Care and Use of Laboratory Animals and were approved by the Purdue Animal Care and Use Committee (PACUC).

### Bacterial strains and growth conditions

Bacterial strains used in this study are listed in Table S2. Strains of *E. coli* were grown in LB and the medium was supplemented with the appropriate antibiotics when necessary. The *L. pneumophila* strain Philadelphia-1 strain Lp02 [61] was the parent of all derivatives used in this study. *L. pneumophila* was grown and maintained on CYE medium as previously described [62]. When necessary antibiotics were included as described [62]. To construct the *sidK* in-frame deletion mutant ZL114, we constructed plasmid pZL886 by cloning two DNA fragments generated by primers PL192/PL193 and PL194/PL195 (Table S4) into *Sac*I/*Sal*I digested pSR47s [61]. The primers were designed so that after deletion, only the first and last 15 amino acids are left in the mutant. pZL886 was introduced into Lp02 and the deletion mutant was obtained by following the standard allelic exchange method [63]. To complement the mutation, we inserted the coding region of *sidK* into pJB908 [40]. In complementation experiments, the vector used for expressing the gene of interest was introduced into the wild type strain or mutants and the bacterial cultures grown to the post-exponential phase as determined by optical density of the cultures (OD_600_ = 3.3-3.8) as well as an increase of bacterial motility.

### Plasmid construction

The plasmids used in this study are listed in Table S3 and the sequences of all primers are in Table S4. Plasmids harboring individual full-length *L. pneumophila* hypothetical genes were in Table S1. The open reading frame of *sidK* and its derivatives were cloned into pEGFPC1 (Clontech) for expression in mammalian cells. A number of vectors, including pGBKT7 (Clontech), p415ADH, p415TEF, p425TEF and p425GPD [39] were used to express *sidK* in yeast either as an untagged form or as GFP fusions (see text for details). To express His_6_-SidK in *L. pneumophila*, we first amplified the multiple cloning site region of pQE30 (Qiagen) with primers QE5′*Eco*RV/QE3′*Xba*I and inserted it into *Ecl*136II/*Xba*I digested pJB908 [40]. to generate pZL507. The *sidK* gene was then inserted into pZL507 as a *Bam*HI/*Xho*I fragment to give pZL1333. cDNA clones coding for relevant subunits of the v-ATPase were amplified from a human kidney cDNA library (Clontech) or from clones purchased from the ATCC. For expression in mammalian cells, *sidK* or each of these genes was inserted into pEGFPC1 (Clontech) or pFlag-CMV (Sigma). The *vatH* gene (pEF-HA-NBP1) was a gift from Dr. M. Peterlin of University of California, San Francisco. The integrity of all genes was verified by sequencing analysis.

### Yeast manipulation and screening of *L. pneumophila* proteins that inhibits yeast growth in neutral pH medium

Yeast strains used were PJ69-4A [64], BY4741 [38] and their derivatives (Table S2). Yeast was grown in YPD medium or in appropriate amino acid dropout minimal media at 30°C. Using a standard protocol [65], we transformed plasmids carrying full-length hypothetical *L. pneumophila* genes [20] into yeast strain PJ69-4A [64] and grew the resulting strains over night in minimal medium of pH 5.5. After diluting at 1∶40 into medium of pH 7.5 buffered with 50 mM MES and 50 mM MOP, the cultures were incubated with vigorous shaking for 24 hrs. Cultures that did not grow to high density were retained for further analysis. For quantitative study of growth, yeast subcultures of 2×10^6^ cells/ml were made in appropriate Dropout medium and cell growth was monitored by measuring the OD_600_ at indicated time points.

To prepare cell lysates for protein analysis, cells from 5 ml overnight cultures were first lysed with a cracking buffer (40 mM Tris-Cl [pH 6.8], 5% SDS, 0.1 mM EDTA, 8 M urea, bromothymol Blue 0.4 mg/ml) with glass beads. Samples were resolved by SDS-PAGE after adding Laemmli buffer.

### Cell culture and transfection

Mouse macrophages were prepared from bone marrow of female A/J mice of 6–10 weeks of age following published protocols [12]. U937 cells were cultured in RPMI medium supplemented with 10% fetal bovine/calf serum (FBS) and 5 mM glutamate, and if needed, the cells were differentiated into macrophages with 50 ng/ml phorbol myristic acid (PMA) as described [12]. 293T cells were cultured in Dulbecco's modified minimum Eagle's medium (DMEM) supplemented with 10% FBS. For transfection, we grew cells to about 80% confluence and transfected them with Lipofectamine 2000 (Invitrogen) following manufacturer's instructions. For growth curve experiments, macrophages were plated into 24-well plates at 2×10^5^ cell per well. For immunoprecipitation and fractionation, about 2×10^7^ cells plated on standard petri dishes were used. Infection was performed at the indicated MOIs as required by the particular experiments.

### Protein expression and purification

To purify GST-SidK, we inserted the predicted *sidK orf* into pGEX-4T-1 (Qiagen) to generate pZL797. *E. coli* strain XL1Blue containing pZL797 was grown in 1 liter LB to OD_600_ of 0.7. After inducing with 0.2 mM IPTG for 6 hrs at 25°C, harvested cells were lysed by passing through a French press twice at 1,500 psi. Cleared supernatant was incubated with glutathione beads (Qiagen) for 2 hrs at 4°C and the beads were washed with 40X bed volume of PBS buffer containing 0.5% Triton X-100. GST-SidK was eluted with PBS containing 10 mM reduced glutathione. When needed, GST tag was removed by Thrombin, a protease that was subsequently removed by benzamidine-Sepharose beads (GE). GST-VatA was purified with a similar procedure.

To purify His_6_-SidK from *L. pneumophila*, we introduced pZL1333 into the non-virulent strain Lp03 [61]. A 50 ml of saturated culture was diluted into 1 liter AYE broth, when the culture reached exponential growth phase (OD_600_ = 0.5), expression of the gene was induced with 0.2 mM IPTG for 16 hrs. Cleared cell lysates were incubated with Ni^2+^-Agrose beads for 2 hrs at 4°C and the beads were washed with 40 times of the bed volume of TBS buffer (50 mM Tris-HCl, 150 mM NaCl, pH 7.4) containing 10 mM imidazole. The protein was eluted with 200 mM imidazole. After dialyzing against TBS to remove imidazole, the protein was further purified by gel filtration with an FPLC system using a Superdex 200 10/300 GL column (GE Healthcare). TBST buffer (50 mM Tris-Cl, 150 mM NaCl, 0.1% Triton-X100, pH 7.4) was used as eluent and the flow rate was set at 0.4 ml/min. The single peak corresponding to the protein was collected, dialyzed in the appropriate buffer for subsequent use. His_6_-Hsp70 was similarly purified from *E. coli*. Protein concentrations were determined by the Bradford assay; the purity of all proteins was more than 95% as assessed by SDS-PAGE followed by Coomassie bright blue staining (Figs. S3 and S7).

### Affinity chromatograph from cell lysates and *in vitro* protein binding

The procedure for affinity pulldown was described elsewhere [20]. Briefly, U937 cells collected from 500 ml culture suspended in 3.0 ml PBS containing 5 mM DTT and protease inhibitors (Roche) were lysed with a glass homogenizer (Wheaton). The lysates were subjected to centrifugation at 10,000×g for 10 min at 4°C to remove unbroken cells and nuclei, the post-nuclear supernatant was added to Affigel beads coated with SidK and incubated for 14 hrs at 4°C. We then washed the beads five times with PBS and dissolved bound proteins with SDS sample buffer. After SDS-PAGE, proteins were visualized by silver staining (Bio-Rad). Individual protein bands retained by beads coated by SidK but not by GST were excised, digested with trypsin, and analyzed by matrix-assisted laser desorption/ionization/mass spectrometry (MALDI/MS) (Taplin Biological Mass Spectrometry Facility, Harvard Medical School).

For GST pull down experiments, 10 µg purified GST or GST-VatA was mixed with 2 µg His_6_-SidK in PBS for 4 hrs at 4°C. After adding 40 µl of 50% pre-washed glutathione beads slurry, binding was allowed to proceed for 1 hr. The beads were then washed 5 times with PBS containing 500 mM NaCl. Retained proteins were detected by immunoblot after SDS-PAGE.

### Coimmunoprecipitation

Twenty-four hrs after transfection, cells were collected and lysed in a lysis buffer (0.2% of NP-40, 50 mM Tris-HCl pH = 7.5, 150 mM NaCl, 1 mM EDTA, 15% glycerol, and protease inhibitors (1 mM Na_3_VO_4_, 1 mM PMSF, 10 µg/ml Aprotinin, 2 µg/ml Leupeptin, 0.7 µg/ml Pepstatin)). After removing debris by centrifugation at 10,000 g for 10 min at 4°C, 2 mg protein (approximately 1 ml) was used for immunoprecipitation by adding the appropriate antibody and 30 µl of 40% protein G-sepharose beads (GE Healthcare). After incubating at 4°C on a rotary shaker for 4 hrs, the beads were washed 5 times with the lysis buffer before being dissolved in Laemmli buffer.

For immunoprecipitation with yeast lysates, cells harvested from 50 ml mid-log phase cultures were digested with Zymolyase, and the resulting spheroplasts were lysed with the lysis buffer used for mammalian cells. The lysates containing approximately 2 mg proteins were incubated with appropriate antibody and protein G sepharose for 16 hrs at 4°C. The beads were removed by washing 5 times with the lysis buffer. In both cases, protein associated with beads were dissolved in Laemmli buffer and resolved by SDS-PAGE. Proteins transferred to nitrocellulose membranes were detected by immunoblot.

### Antibodies and Western blot

Antisera against Legionella, ICDH (isocitrate dehydrogenase) were described in an early study [4]. SidK cleaved from GST-SidK were used as an antigen to produce a specific antibody following a standard protocol (Pocono Rabbit Farm and Laboratory, Canadensis, PA). GFP antibody was prepared similarly with purified His_6_-GFP. When necessary, antibodies were affinity-purified against the antigens covalently coupled to an Affigel matrix (Bio-Rad) using standard protocols [66]. Monoclonal or polyclonal antibodies against Flag, Vma1, Vma2, PGK (3-phosphoglycerate kinase), VatA and Hsp70 were purchased from Sigma, Invitrogen, Abcam and Santa Cruz Biotechnology (sc-65521), respectively.

For Western blots, samples resolved by SDS-PAGE were transferred onto nitrocellulose membranes. After blocking with 4% milk in PBS buffer containing 0.2% Tween 20, membranes were incubated with the appropriate primary antibody: anti-SidK, 1∶2,500; anti-VatA, 1∶10,000; anti-Vma1, 1∶1,000; anti-Vma2, 1∶1,000; anti-GFP, 1∶50,000; anti-PGK, 1∶ 2000; anti-ICDH, 1∶5,000; anti-Hsp70, 1∶2000. Horseradish peroxidase conjugated secondary antibodies and enhanced bioluminescence reagents were used to detect the signals (Pierce, Rockford, IL). Alternatively, membranes were incubated with an appropriate IRDye infrared secondary antibody (Li-Cor's Biosciences, Lincoln, Nebraska, USA) and the signals were detected, and if necessary, the intensity of the bands are quantitiated by using the Odyssey infrared imaging system.

### Preparation of yeast vacuolar membrane vesicles

Yeast vacuolar membrane vesicles were prepared according to the standard method [45] with some modification. Briefly, exponentially growing yeast cells (O.D. = 0.6) were harvested, washed twice with distilled water and digested with zymolyase at 30°C for 90 min in 1 M sorbitol. The spheroplasts were resuspended in 7 volumes of Buffer A (10 mM MES/Tris (pH 6.9), 12% Ficoll 400, and 0.1 mM MgCl_2_), homogenized in a loosely fitting Dounce homogenizer (Wheaton) with 20 strokes, and centrifuged in a swinging bucket rotor at 4,500 g for 10 min. The supernatants were transferred to new centrifuge tubes, and buffer A was layered on the top. After centrifugation at 51,900×g for 40 min, the white layer on the top was collected and resuspended in Buffer A with a homogenizer, and Buffer B (10 mM MES/Tris (pH 6.9), 8% Ficoll 400, and 0.5 mM MgCl_2_) was layered on the top. After similar centrifugation, vacuoles free from lipid granules or other membranous organelles were collected from the top of the tube. Vacuolar membrane vesicles were prepared by diluting the purified vacuoles in a vesicle buffer (10 mM MES/Tris (pH 6.9), 5 mM MgCl_2_, and 25 mM KC1).

### V-ATPase-mediated ATP hydrolysis assay

Equal amount of vacuolar membranes in ATPase buffer (10 mM HEPES, 5 mM MgCl_2_, 125 mM KCl, pH = 7.0) were preincubated for 40 min at room temperature with or without the testing chemicals or proteins. To test the effect of antibody, purified antibody was added to the reactions for 20 min before the addition of ATP. BSA dissolved in the same buffer as that of SidK was used as a negative control. The reaction was initiated by adding 1 mM of ATP, and samples were withdrawn at indicated time points to measure the production of inorganic phosphate using the malachite green method [46]. Briefly, the malachite green reagent was made of 2 volumes of 0.0812% malachite green, 1 volume of 5.72% ammonium molybdate dissolved in 6 M HCl, 1 volume of 2.32% polyvinyl alcohol and 2 volumes of distilled water. 90 µl of the malachite green reagent was added to 10 µl samples withdrawn at indicated time points. The reactions were allowed to proceed for 2 min and were terminated with 1/10 volume of 34% sodium citrate. After incubation for another 20 min, absorbance at OD_620_ nm was measured. A standard curve simultaneously obtained with a series of phosphate solutions of known concentrations was used to determine the amount of phosphate released by the membranes.

### Proton translocation activity

The ATP-driven proton transport activity was assayed by measuring the uptake of proton in the yeast membrane vesicles using acridine orange (AO) quenching assay [67]. Purified vacuolar membrane vesicles were diluted in AO buffer (5 mM HEPES, pH = 7.0, 5 mM MgCl_2_, 150 mM KCl, 6 µM AO), and preincubated for 40 min at room temperature with or without the testing chemicals or proteins. The reaction was initiated by adding 2 mM of ATP and quickly mixed. The quenching of acridine orange was monitored by the Spex FluoroMax 3 spectrofluorometer (Jobin Yvon) with excitation at 493 nm and emission at 525 nm.

### Measurement of vacuolar pH

The pH of *L. pneumophila*-containing phagosomes was determined by fluorescence ratio imaging using 5(6)-carboxyfluorescein-*N*-hydroxysuccinamide ester (FLUOS, Fluka) stained *L. pneumophila* as previously described [30] with the modifications detailed below. For labeling, *L. pneumophila* were cultured to the post-exponential phase, defined by motility and OD_600_ = 3.6 - 4.6, washed once with 100 mM potassium phosphate buffer, pH 8.0, and then incubated for 20 min at room temperature with 0.8 mg/ml FLUOS in 4% DMSO in 100 mM potassium phosphate, pH 8.0. This treatment did not affect viability of bacteria as determined by quantifying colony formation.

We infected macrophages plated on 24 mm coverslips with an MOI of ∼10. Infections were synchronized by washing infected cells 4 times with RPMI/FBS 60 minutes after uptake. Following an additional 70–180 minutes of incubation, we washed the monolayers 3 times with 37°C Ringers Buffer (RB; 55 mM NaCl, 5 mM KCl, 2 mM CaCl_2_, 1 mM MgCl_2_, 2 mM NaH_2_PO_4_, 10 mM HEPES, and 10 mM glucose, pH 7.2), and placed the samples at a 37°C chamber with 1 ml RB and visualized on an Olympus IX70 inverted microscope. Images were acquired from the attached CoolSNAP HQ2 14-bit CCD camera (Photometrics) and subsequently analyzed using Metamorph Premier v6.3 software (Molecular Devices).

Fluorescence images were obtained at excitation wavelengths of 492 nm and 436 nm and corrected for bias, shading, and background. Individual bacteria were masked by manual thresholding using an addition image of both wavelengths. The mask was then applied to each original corrected image, and the average fluorescence intensity over the masked area of each particle was determined at each wavelength. The pH of each *L. pneumophila*-containing phagosome was calculated from the ratio of the fluorescence intensity at an excitation wavelength of 492 nm to the intensity at excitation 436 nm. The fluorescence intensity ratios from two independent experiments were converted to pH using a single standard curve of quartic function. The standard curve was established using FLUOS-labeled bacteria immobilized on a coverslip coated with poly-(L)-lysine. Samples were processed as above, with greater than 130 bacteria analyzed per pH at 10 incremental values ranging from pH 3.5 to 8.5 in clamping buffer (130 mM KCl, 1 mM MgCl_2_, 15 mM HEPES, 15 mM MES).

Only intact rod-shaped bacteria were evaluated, with greater than 50 bacteria analyzed per coverslip in each of two independent experiments. To verify that *sidK* did not affect lysosomal degradation, the fraction of wild-type and *sidK* mutant bacteria that were degraded was quantified 1 h post-infection by fixed immunofluorescence microscopy as previously described [68] (Fig. S2). Heat killed (80°C 20 minutes) wild-type bacteria served as a control for particles that trafficked to an acidic compartment [69].

### Protein loading, lysosomal digestion of bacteria and phagosomal pH evaluation

We delivered recombinant proteins into macrophages by the syringe loading method [52] with some modifications: Briefly, cells were washed and collected in ice cold Dulbecco's PBS (DPBS) (Cellgro) by centrifugation (200 g 5 min). We then washed the cells twice with 37°C DPBS containing 1.2 mM CaCl_2_ before adding 200 µl warm loading solutions (DPBS containing 1.2 mM CaCl_2_ and the protein to be loaded at 0.4 mg/ml) to cell pellet containing 5×10^6^ cells. A P-200 micropipettor (Rainin) set at 100 µl was used to pipett the cell suspension for 100 times at 37°C. After pipetting, the mixture was incubated at 37°C for 2 min. After washing twice with warm DPBS containing 1.2 mM CaCl_2_, cells were seeded onto glass coverslips in 24-well plates with a density of 4×10^5^ per well and were incubated at 37°C for 12–16 hrs.

The bactericidal activity of macrophages loaded with different proteins was measured according to a published method [54] with minor modifications. Cells of *E. coli* strain XL1-Blue expressing the mCherry RFP or GFP were added to macrophages at an MOI of 10 for 1 hr at 37°C. The culture supernatant was replaced with fresh tissue culture medium containing 100 µg/ml gentamicin to kill extracellular bacteria. After 1 hr of incubation, the medium was replaced with fresh medium containing 10 µg/ml gentamicin. At indicated time points, cells were washed extensively (5x) with warm PBS and lysed with 0.02% saponin. The lysates were plated on LB plates and colonies were counted after overnight incubation at 37°C.

For the staining with fluorescein dextran, 10 kD fluorescent dextran (Invitrogen) was added to the cells to 0.2 mg/ml; the pH insensitive 10 kD Cascade Blue dextran (Invitrogen) was added at 0.2 mg/ml as a loading control. After incubating at 37°C for1 hr, the cells were washed 5 times and incubated at 37°C for an additional 4 hrs before being imaged. For the staining with LysoRed (LysoTracker Red DND-99, Invitrogen), cells of an *E. coli* strain expressing GFP were added to macrophage monolayer at an MOI of 20 for 1 hr at 37°C. After incubating with a medium containing 100 µg/ml gentamicin for 1 hr and then a medium containing 10 µg/ml gentamicin for an additional 8 hrs, a medium containing 50 nM LysoRed was added to the samples for 15 min. Cells were washed 3 times with fresh medium and subjected to imaging analysis immediately under an Olympus X-81 fluorescence microscope. All images were acquired with identical digital imaging parameters (objectives, exposure duration, contrast ratios, etc.) and were similarly processed using the IPlab software package (BD Biosciences).

### Gene accession number

The gene described in this manuscript is lpg0968 with an accession number of YP_095002 in the Genebank.

## Supporting Information

Table S1 *L. pneumophila* proteins tested for conferring yeast sensitivity to neutral pH.
(0.08 MB DOC)

Table S2 Bacterial and yeast strains used in this study.
(0.17 MB DOC)

Table S3 Plasmids used in this study.
(0.10 MB DOC)

Table S4 Primers used in this study.
(0.07 MB DOC)

Figure S1 Deletion of *sidK* did not affect intracellular growth of *L. pneumophila*. Indicated bacterial strains grown to post-exponential phase were used to infect mouse macrophages (A) or *Dictyostelium discoideum* (B). Infections were synchronized 1 h after uptake and the total bacterial counts at indicated time points were determined by lysing infected cells with 0.02% saponin and plating appropriately diluted lysates on CYE plates. Data shown are one representative experiment done in triplicates. Similar results are obtained in more than three independent experiments.
(0.16 MB PDF)

Figure S2 Vacuoles containing the *sidK* deletion mutant maintain a neutral luminal pH. Mouse macophages were infected with indicated *L. pneumophila* strains for 2 hours and vacuolar pH of the phagosomes was measured as described in Materials and Methods (A). The integrity of the internalized bacteria also was examined (B). More than 50 vacuoles were scored for each coverslip. Similar results were obtained in two independent experiments.
(0.25 MB PDF)

Figure S3 Purification of SidK from *L. pneumophila*. A derivative of the avirulent strain Lp03 containing pZL1333 that direct the expression of His_6_-SidK was grown in AYE broth and the expression of the protein was induced with IPTG for 16 hours. His_6_-SidK was first purified by a Ni^2+^ column followed by FPLC with an AKTA system. Fractions containing the protein were pooled and dialysed in TBS buffer. Image shown are different amount of His_6_-SidK resolved SDS-PAGE and stained by Coomassie bright blue staining. Lanes: 1, 1 µg; 2, 2 µg; 3, 4 µg; 4, 8 µg.
(0.23 MB PDF)

Figure S4 Interactions between v-ATPase and SidK deletion mutants in yeast. The indicated SidK deletionmutants (A) were expressed in yeast as GFP fusions. Total cell lysates were subjected to co-immunopreciitation with a GFP specific antibody and the presence of Vma1 and Vma2 in the precipitates were detected by immunoblot (B). The presence of Vma1 and Vma2 in the cell lyates was detected (C, upper panel) and the expression of the SidK truncations was evaluated with the GFP specific antibody (B, lower panel).
(0.26 MB PDF)

Figure S5 Expression of sidK mutants in yeast. Indicated mutants were cloned into p425GPD, a start codon (ATG) was added to N-terminal deletion mutant. Samples were processed as described for Fig. 1 and proteins were detected with an anti-SidK antibody.
(0.23 MB PDF)

Figure S6 The SidK specific antibody did not affect SidK activity. v-ATPase assays were performed as described in Fig. 7A. 0.4 µM of SidK was used in each reaction. Indicated amount of antibody specific for SidJ or SidK was added 20 min after the addition of SidK. Reactions were allowed to proceed for 60 min and the release of free phosphate was measured.
(0.08 MB PDF)

Figure S7 SidK does not affect ATPase activity of Hsp70. Mammalian Hsp70 was purified from *E. coli* as a His_6_-tagged protein (A). 0.5 ug of purified protein was incubated with 2 mM ATP and the indicated compounds or proteins for various periods of time (X-axis). Hydrolysis of ATP was monitored by measuring released free phosphate with malachite green (B). Similar results were obtained in two experiments done in triplicates. Concentrations of testing materials: BSA, 1 µM; SidK, 1 µM; Vanadate, 1 mM; EDTA, 10 mM.
(0.23 MB PDF)
