# Supplementary material for: Inhibition of Host Vacuolar H+-ATPase Activity by a Legionella pneumophila Effector
Source: PLoS Pathog. 2010 Mar 19;6(3):e1000822. doi: 10.1371/journal.ppat.1000822 (PMC2841630; doi:10.1371/journal.ppat.1000822)
Supplement: Table S1 — L. pneumophila proteins tested for conferring yeast sensitivity to neutral pH. (0.08 MB DOC) [file ppat.1000822.s001.doc]

Table S1 *L. pneumophila* proteins tested for conferring yeast sensitivity to neutral pH.

| Number | Lpg number | Number | Lpg number | Number | Lpg number |
| --- | --- | --- | --- | --- | --- |
| 1 | lpg0008 | 34 | lpg1496 | 67 | lpg2424 |
| 2 | lpg0012 | 35 | lpg1602 | 68 | lpg2464 |
| 3 | lpg0086 | 36 | lpg1666 | 69 | lpg2523 |
| 4 | lpg0096 | 37 | lpg1683 | 70 | lpg2526 |
| 5 | lpg0149 | 38 | lpg1684 | 71 | lpg2527 |
| 6 | lpg0150 | 39 | lpg1687 | 72 | lpg2529 |
| 7 | lpg0196 | 40 | lpg1705 | 73 | lpg2568 |
| 8 | lpg0269 | 41 | lpg1717 | 74 | lpg2582 |
| 9 | lpg0284 | 42 | lpg1751 | 75 | lpg2603 |
| 10 | lpg0360 | 43 | lpg1809 | 76 | lpg2622 |
| 11 | lpg0634 | 44 | lpg1949 | 77 | lpg2627 |
| 12 | lpg0642 | 45 | lpg1958 | 78 | lpg2637 |
| 13 | lpg0696 | 46 | lpg1963 | 79 | lpg2719 |
| 14 | lpg0771 | 47 | lpg1969 | 80 | lpg2744 |
| 15 | lpg0774 | 48 | lpg1979 | 81 | lpg2745 |
| 16 | lpg0788 | 49 | lpg1986 | 82 | lpg2758 |
| 17 | lpg0944 | 50 | Lpg2050 | 83 | lpg2759 |
| 18 | lpg0968 | 51 | lpg2148 | 84 | lpg2804 |
| 19 | lpg0969 | 52 | lpg2155 | 85 | lpg2826 |
| 20 | lpg0974 | 53 | lpg2160 | 86 | lpg2844 |
| 21 | lpg1073 | 54 | lpg2161 | 87 | lpg2853 |
| 22 | lpg1109 | 55 | lpg2166 | 88 | lpg2856 |
| 23 | lpg1129 | 56 | lpg2220 | 89 | lpg2864 |
| 24 | lpg1145 | 57 | lpg2223 | 90 | lpg2877 |
| 25 | lpg1148 | 58 | lpg2239 | 91 | lpg2879 |
| 26 | lpg1183 | 59 | lpg2242 | 92 | lpg2912 |
| 27 | lpg1227 | 60 | lpg2248 | 93 | lpg2913 |
| 28 | lpg1228 | 61 | lpg2313 | 94 | lpg2939 |
| 29 | lpg1234 | 62 | lpg2399 | 95 | lpg2959 |
| 30 | lpg1265 | 63 | lpg2400 | 96 | lpg2976 |
| 31 | lpg1329 | 64 | lpg2410 | 97 | lpg3000 |
| 32 | lpg1354 | 65 | lpg2422 |  |  |
| 33 | lpg1481 | 66 | lpg2423 |  |  |
