# Supplementary material for: Inhibition of Host Vacuolar H+-ATPase Activity by a Legionella pneumophila Effector
Source: PLoS Pathog. 2010 Mar 19;6(3):e1000822. doi: 10.1371/journal.ppat.1000822 (PMC2841630; doi:10.1371/journal.ppat.1000822)
Supplement: Table S2 — Bacterial and yeast strains used in this study. (0.17 MB DOC) [file ppat.1000822.s002.doc]

| Table S2 Bacterial and yeast strains used in this study | |  |
| --- | --- | --- |
| Strains | Genotype, relevant markers | Reference |
| *E. coli* |  | Stratagene |
| XL1-Blue | *recA1* endA1 *gyrA*96 *thi-1* *hsd*R17 *sup*E44 *relA*1 *lac* [*F*' *proAB lacI*^q^*ZM15* Tn*10*(Tet^r^)] |
| DH5α(λpir) | supE44 d*lacU169*(φ80*lacZ*Δ*M15*) *hsdR17 recA1 endA1 gyrA96 thi-1 relA1 pir tet*::*Mu recA* | Our collection |
| *L. pneumophila* |  |  |
| Lp02 | Philadelphia-1 *rpsL hsdR thyA* | [1] |
| ZL25 | LP02*∆sidC* | [2] |
| ZL25(pZL199) | LP02*∆sidC*(pZLSidC) | [3] |
| ZL25(pZL204) | LP02*∆sidC*(pSidC∆C100) | [3] |
| ZL370 | LP02*∆sidC*(pZL204::K) | This study |
| ZL371 | LP03(pZL204::K) | This study |
| Lp03 | Lp02(*dotA*^-^) | [1] |
| Lp02(pJB908) | Lp02(pJB908) | [4] |
| ZL14 | Lp03(pJB908) | [4] |
| ZL114 | Lp02*∆sidK* | This study |
| ZL115 | Lp02*∆sidK* (pJB908) | This study |
| ZL207 | Lp02*∆sidK* (pJB908::K) | This study |
| ZL156 | Lp02(pJB2581::K) | This study |
| ZL157 | Lp03(pJB2581::K) | This study |
| ZL269 | Lp03∆K , pZL507::K | This study |
|  |  |  |
| Yeast |  |  |
| PJ69-4A | *MAT***a** *GALI-HIS3 GAL2-ADE8 GAL7-lacZ leu2 ura3 his3 gal4 gal80* | [5] |
| BY4741 | *MATa his3 1 leu2 0 met15 0 ura3 0* | [6] |
| BY4741 | *vma1∆::KanMX* | [6] |
| BY4741 | *Vma2∆::KanMX* | [6] |
| BY4741 | *Vma4∆::KanMX* | [6] |
| BY4741 | *Vma5∆::KanMX* | [6] |
| BY4741 | *Vma7∆::KanMX* | [6] |
| BY4741 | *Vma8∆::KanMX* | [6] |
| BY4741 | *Vma10∆::KanMX* | [6] |
| BY4741 | *Vma13∆::KanMX* | [6] |
|  |  |  |
|  |  |  |

Reference

1. Berger KH, Isberg RR (1993) Two distinct defects in intracellular growth complemented by a single genetic locus in Legionella pneumophila. Mol Microbiol 7: 7-19.

2. Luo ZQ, Isberg RR (2004) Multiple substrates of the Legionella pneumophila Dot/Icm system identified by interbacterial protein transfer. Proc Natl Acad Sci U S A 101: 841-846.

3. VanRheenen SM, Luo ZQ, O'Connor T, Isberg RR (2006) Members of a Legionella pneumophila family of proteins with ExoU (phospholipase A) active sites are translocated to target cells. Infect Immun 74: 3597-3606.

4. Liu Y, Luo ZQ (2007) The Legionella pneumophila effector SidJ is required for efficient recruitment of endoplasmic reticulum proteins to the bacterial phagosome. Infect Immun 75: 592-603.

5. James P, Halladay J, Craig EA (1996) Genomic libraries and a host strain designed for highly efficient two-hybrid selection in yeast. Genetics 144: 1425-1436.

6. Winzeler EA, Shoemaker DD, Astromoff A, Liang H, Anderson K, et al. (1999) Functional characterization of the S. cerevisiae genome by gene deletion and parallel analysis. Science 285: 901-906.

7. Bardill JP, Miller JL, Vogel JP (2005) IcmS-dependent translocation of SdeA into macrophages by the Legionella pneumophila type IV secretion system. Mol Microbiol 56: 90-103.

8. Mumberg D, Muller R, Funk M (1995) Yeast vectors for the controlled expression of heterologous proteins in different genetic backgrounds. Gene 156: 119-122.
