# Supplementary material for: Inhibition of Host Vacuolar H+-ATPase Activity by a Legionella pneumophila Effector
Source: PLoS Pathog. 2010 Mar 19;6(3):e1000822. doi: 10.1371/journal.ppat.1000822 (PMC2841630; doi:10.1371/journal.ppat.1000822)
Supplement: Table S3 — Plasmids used in this study. (0.10 MB DOC) [file ppat.1000822.s003.doc]

| Table S3 Plasmids used in this study | | |
| --- | --- | --- |
|
| Plasmid | Relevant phenotypes | Sources |
| pQE30 | Amp | Qiagen |
| pJB908 | Amp, *thy*^+^ | [7] |
| pJB2581 | Cm, Amp | [7] |
| pGEX-4T-1 | Amp | Qiagen |
| pZL204 | Amp, *thy*^+^ SidC*∆C100* on pJB908 | [2] |
| pZL507 | For expression His_6_-tagged protein *L. pneumophila* | This study |
| pEGFPC-1 | For expressing C-terminal GFP fusion proteins | Clontech |
| p415-ADH | Amp, *leu*^+^, ADH promoter | [8] |
| p415-TEF | Amp, *leu*^+^, TEF promoter | [8] |
| p425-GPD | Amp, *leu*^+^, GPD promoter | [8] |
| p425-TEF | Amp, *leu*^+^, TEF promoter | [8] |
| pGBKT7 | Km, *trp*^+^ | Clontech |
| pZL796 | pZL204::K, BamHI, XbaI | This study |
| pZL797 | pGEX-4T-1::K, BamHI, XbaI | This study |
| pZL812 | pGBKT7::K, EcoRI, BamHI | This study |
| pZL863 | pEGFPc1::K, BglII, EcoRI | This study |
| pZL864 | pCMVFlagM-VatA, BglII, SalI | This study |
| pZL866 | pSR47s::SidK-knockout | This study |
| pZL1048 | pQE-30::Hsp70, BamHI/SalI | This study |
| pZL1109 | pCMVFlagM::VatC, BglII, SalI | This study |
| pZL1264 | pEGFPc1::K-C1, BglII/BamHI, PstI | This study |
| pZL1265 | pEGFPc1::K-C2, BglII/BamHI, PstI | This study |
| pZL1266 | pEGFPc1::K-C3, BglII/BamHI, PstI | This study |
| pZL1272 | pEGFPc1::K-N2, BglII/BamHI, EcoRI | This study |
| pZL1273 | pEGFPc1::K-N1, BglII/BamHI, EcoRI | This study |
| pZL1281 | pEGFPc1::K-C4, BglII/BamHI, EcoRI | This study |
| pZL1282 | pEGFPc1::K-C5, BglII/BamHI, EcoRI | This study |
| pZL1283 | pEGFPc1::K-N30, BglII/BamHI, EcoRI | This study |
| pZL1333 | pZL507::K, BamHI, SalI/XhoI | This study |
| pZL1463 | p415-ADH::K, BamHI, SalI/XhoI | This study |
| PZL1508 | p416-ADH::Flag-Vma5, BamHI, SalI/XhoI | This study |
| pZL1517 | p415-ADH::GFP-K, BamHI/BglII, SalI/XhoI | This study |
| pZL1518 | p415-ADH::GFP-KN30, BamHI/BglII, SalI/XhoI | This study |
| pZL1519 | p415-ADH::GFP-KC2, BamHI/BglII, PstI | This study |
| pZL1520 | p415-ADH::GFP-KC3, BamHI/BglII, PstI | This study |
| pZL1521 | p415-ADH::GFP-KC4, BamHI/BglII,PstI | This study |
| pZL1543 | p415-ADH::GFP-KN1, BamHI/BglII, SalI/XhoI | This study |
| pZL1544 | p415-ADH::GFP-KN2, BamHI/BglII, SalI/XhoI | This study |
| pZL1546 | p415-ADH::GFP-KC5, BamHI/BglII, PstI | This study |
| pZL1572 | p415-ADH::GFP, BamHI/BglII, SalI | This study |
| pZL1585 | p415-TEF::K, BamHI, SalI/XhoI | This study |
| pZL1586 | p425-GPD::K, BamHI, SalI/XhoI | This study |
| pZL1587 | p425-TEF::K, BamHI, SalI/XhoI | This study |
| pZL1593 | p425-GPD::KN1, BamHI, SalI/XhoI | This study |
| pZL1594 | p425-GPD::KC1, BamHI, SalI/XhoI | This study |
| pZL1596 | p425-GPD::KC3, BamHI, SalI/XhoI | This study |
| pZL1628 | p425-GPD::KN30, BamHI, PstI | This study |
| pZL1645 | p416-ADH::Flag-Vma4, BamHI, SalI/XhoI | This study |
|  |  |  |

Referecne

1. Berger KH, Isberg RR (1993) Two distinct defects in intracellular growth complemented by a single genetic locus in Legionella pneumophila. Mol Microbiol 7: 7-19.

2. Luo ZQ, Isberg RR (2004) Multiple substrates of the Legionella pneumophila Dot/Icm system identified by interbacterial protein transfer. Proc Natl Acad Sci U S A 101: 841-846.

3. VanRheenen SM, Luo ZQ, O'Connor T, Isberg RR (2006) Members of a Legionella pneumophila family of proteins with ExoU (phospholipase A) active sites are translocated to target cells. Infect Immun 74: 3597-3606.

4. Liu Y, Luo ZQ (2007) The Legionella pneumophila effector SidJ is required for efficient recruitment of endoplasmic reticulum proteins to the bacterial phagosome. Infect Immun 75: 592-603.

5. James P, Halladay J, Craig EA (1996) Genomic libraries and a host strain designed for highly efficient two-hybrid selection in yeast. Genetics 144: 1425-1436.

6. Winzeler EA, Shoemaker DD, Astromoff A, Liang H, Anderson K, et al. (1999) Functional characterization of the S. cerevisiae genome by gene deletion and parallel analysis. Science 285: 901-906.

7. Bardill JP, Miller JL, Vogel JP (2005) IcmS-dependent translocation of SdeA into macrophages by the Legionella pneumophila type IV secretion system. Mol Microbiol 56: 90-103.

8. Mumberg D, Muller R, Funk M (1995) Yeast vectors for the controlled expression of heterologous proteins in different genetic backgrounds. Gene 156: 119-122.
