# Supplementary material for: Inhibition of Host Vacuolar H+-ATPase Activity by a Legionella pneumophila Effector
Source: PLoS Pathog. 2010 Mar 19;6(3):e1000822. doi: 10.1371/journal.ppat.1000822 (PMC2841630; doi:10.1371/journal.ppat.1000822)
Supplement: Table S4 — Primers used in this study. (0.07 MB DOC) [file ppat.1000822.s004.doc]

| Table S4 Primers used in this study | |  |
| --- | --- | --- |
|  | |  |
| Primer | Sequence (Restriction enzyme sites are underlined) | Note |
| QE3 | CTCTTCTAGAGTTCTTTACGATGCCATTGGG | QE3'XbaI |
| QE5 | CTAGGATATCCGGAAATGTTGAATACTCATAC | QE5'EcoRV |
| PL130 | CTGGGATCCTTGTCTTTTATCAAGGTAGG | sidK5’BamHI |
| PL131 | CTGTCTAGATTAAAGGCTTAGGCTTTCTT | sidK3’XbaI |
| PL142 | CTGGAATTCTTGTCTTTTATCAAGGTAG | sidK5’EcoRI |
| PL143 | CGCGGATCCTTAAAGGCTTAGGCTTTC | sidK3’BamHI |
| PL177 | CTAGAGATCTATGGATTTTTCCAAGCTACC | VatA 5'BglII |
| PL178 | CCTGGTCGACCTTCTAATCTTCAAGGCTAC | VatA 3'SalI |
| PL192 | CCTGGTCGACGCAAAGAACCGGGAAGAG | sidK up SalI knock-out |
| PL193 | CTAGGATCCCCAGAACCTGTGTGGATC | sidK up BamHI knock-out |
| PL194 | CTAGGATCCAGCCACCCATTTTTATACC | sidK down BamHI knockout |
| PL195 | CTAGAGCTCGCTTTATCTCTGTTTCAGG | sidK down SacI knock-out |
| PL208 | CTAGAGATCTAACTGAGTTCTGGCTTATAATCTG | VatC1FBglII |
| PL209 | CCTGGTCGACTCACTTGAATTCCAGCAAGTTG | VatC1RSalI |
| PL312 | CTGGGATCCGCCAAAGCCGCGGCGATCGG | Hsp70 5’ BamHI |
| PL313 | CTGGTCGACCTAATCTACCTCCTCAATGG | Hsp70 3’ SalI |
| PL391 | ACCGGATCCATGTCTTTTATCAAGGTAGG | sidK 5’BamHI |
| PL494 | GCCCTGCAGTTAAAGGCTTAGGCTTTCTTCC | sidK 3’ PstI |
| PL495 | AGGCTCGAGTTAAAGGCTTAGGCTTTCTTCCTG | sidK 3’ XhoI |
| PL526 | GCCGGATCCATGAGATGCATGCAAACTATAGATC | sidK-N30 5’ BamHI |
| PL528 | CCAGGATCCATGGTTTCATCAATTAGTCCC | sidK-N100 5’ BamHI |
| PL416 | GCCGGATCCATGATAGATGAACAATATCACCTG | sidK-N200 5' BamHI |
| PL529 | ACCGGATCCATGATCAGGGAAATTGAAGTG | sidK-N300 5’ BamHI |
| PL530 | CGGCTGCAGTTACCCAAGAAGCTAATACAATTC | sidK-C100 3’ PstI |
| PL531 | CAACTGCAGTTACTATGGAGTAATATCTTC | sidK-C200 3’ PstI |
| PL532 | AGGCTGCAGTTAATCCAGGCCAAATTTACC | sidK-C300 3’ PstI |
| PL533 | GTGCTGCAGTTATGAAACAAGCCGCAGCAATTC | sidK-C400 3’ PstI |
| PL534 | GTCCTGCAGTTATGAAACAAGCCGCAGCAATTC | sidK-C500 3’ PstI |
| PL556 | GGCGGATCCATGGACTACAAAGACGATGACGA | M2-Vma5 5'BamHI |
|  | CAAGGCTACTGCGTTATATACTGC |  |
| PL564 | GCACTCGAGTTATTCGTTAGCCATTGCGAGC | sidK-C4 3'XhoI |
| PL565 | CGGCTCGAGTTAATCCAGGCCAAATTTACC | sidK-C3 3'XhoI |
| PL571 | TCAAGATCTATGGTGAGCAAGGGCGAGGAG | GFP 5’BglII |
| PL572 | GACGGATCCATGGACTACAAAGACGATGACGAC | M2-Vma4 5'BamHI |
|  | AAGTCCTCCGCTATTACTGCTTTGAC |  |
| PL574 | GCCGTCGACTCAATCAAAGAACTTTCTTG | Vma4 3'XhoI |
