# Supplementary material for: Inhibition of Host Vacuolar H+-ATPase Activity by a Legionella pneumophila Effector
Source: PLoS Pathog. 2010 Mar 19;6(3):e1000822. doi: 10.1371/journal.ppat.1000822 (PMC2841630; doi:10.1371/journal.ppat.1000822)
Supplement: Figure S1 — Deletion of sidK did not affect intracellular growth of L. pneumophila. Indicated bacterial strains grown to post-exponential phase were used to infect mouse macrophages (A) or Dictyostelium discoideum (B). Infections were synchronized 1 h after uptake and the total bacterial counts at indicated time points were determined by lysing infected cells with 0.02% saponin and plating appropriately diluted lysates on CYE plates. Data shown are one representative experiment done in triplicates. Similar results are obtained in more than three independent experiments. (0.16 MB PDF) [file ppat.1000822.s005.pdf]

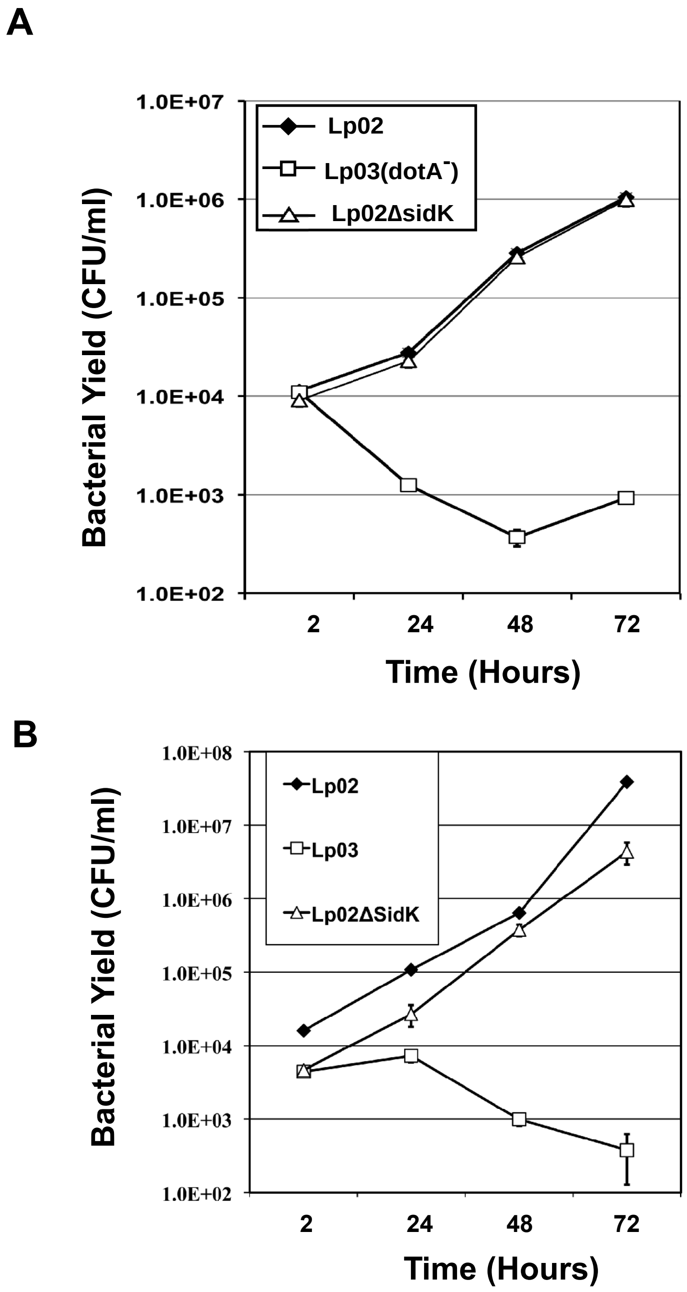

Fig. S1 Deletion of *sidK* did not affect intracellular growth of *L. pneumophila*. Indicated bacterial strains grown to post-exponential phase were used to infect mouse macrophages (**A**) or *Dictyostelium discoideum* (**B**). Infections were synchronized 1 h after uptake and the total bacterial counts at indicated time points were determined by lysing infected cells with 0.02% saponin and plating appropriately diluted lysates on CYE plates. Data shown are one representative experiment done in triplicates. Similar results are obtained in more than three independent experiments.
