# Supplementary material for: Inhibition of Host Vacuolar H+-ATPase Activity by a Legionella pneumophila Effector
Source: PLoS Pathog. 2010 Mar 19;6(3):e1000822. doi: 10.1371/journal.ppat.1000822 (PMC2841630; doi:10.1371/journal.ppat.1000822)
Supplement: Figure S2 — Vacuoles containing the sidK deletion mutant maintain a neutral luminal pH. Mouse macophages were infected with indicated L. pneumophila strains for 2 hours and vacuolar pH of the phagosomes was measured as described in Materials and Methods (A). The integrity of the internalized bacteria also was examined (B). More than 50 vacuoles were scored for each coverslip. Similar results were obtained in two independent experiments. (0.25 MB PDF) [file ppat.1000822.s006.pdf]

A

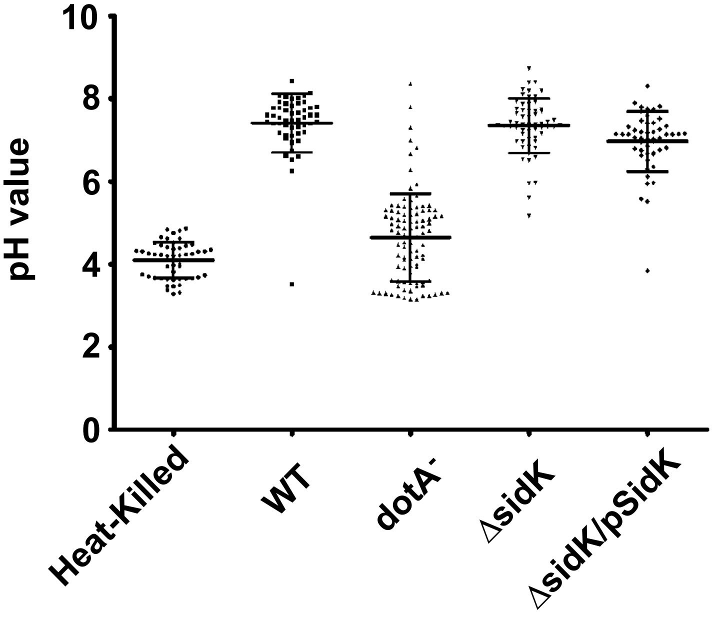

B

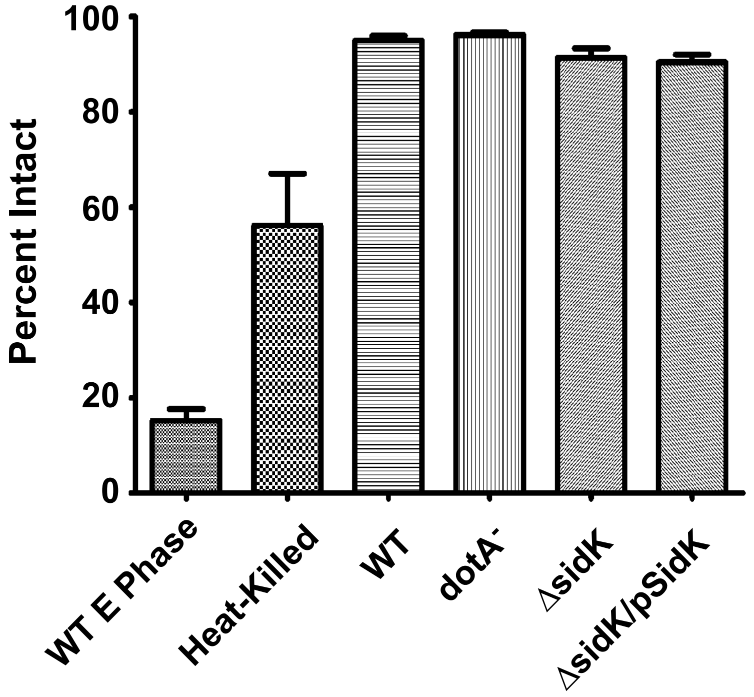

Fig. S2 Vacuoles containing the sidK deletion mutant maintain a neutral luminal pH. Mouse macophages were infected with indicated L. pneumophila strains for 2 hours and vacuolar pH of the phagosomes was measured as described in Materials and Methods (**A**). The integrity of the internalized bacteria also was examined (**B**). More than 50 vacuoles were scored for each coverslip. Similar results were obtained in two independent experiments.
