# Supplementary material for: Inhibition of Host Vacuolar H+-ATPase Activity by a Legionella pneumophila Effector
Source: PLoS Pathog. 2010 Mar 19;6(3):e1000822. doi: 10.1371/journal.ppat.1000822 (PMC2841630; doi:10.1371/journal.ppat.1000822)
Supplement: Figure S3 — Purification of SidK from L. pneumophila. A derivative of the avirulent strain Lp03 containing pZL1333 that direct the expression of His6-SidK was grown in AYE broth and the expression of the protein was induced with IPTG for 16 hours. His6-SidK was first purified by a Ni2+ column followed by FPLC with an AKTA system. Fractions containing the protein were pooled and dialysed in TBS buffer. Image shown are different amount of His6-SidK resolved SDS-PAGE and stained by Coomassie bright blue staining. Lanes: 1, 1 µg; 2, 2 µg; 3, 4 µg; 4, 8 µg. (0.23 MB PDF) [file ppat.1000822.s007.pdf]

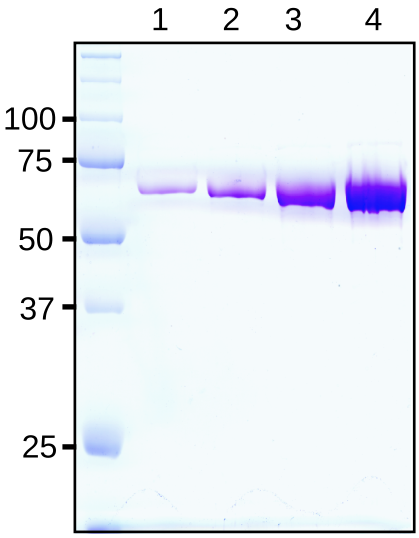

Fig. S3 Purification of SidK from L. pneumophila. A derivative of the avirulent strain Lp03 containing pZL1333 that direct the expression of His_6-SidK was grown in AYE broth and the expression of the protein was induced with IPTG for 16 hours. His_6-SidK was first purified by a Ni^{2+}column followed be FPLC with an AKTA system. Fractions containing the protein were pooled and dialysed in TBS buffer. Image shown are different amount of His_6-SidK resolved SDS-PAGE and stained by Coomassie bright blue staining. Lanes: 1, 1 μg; 2, 2 μg; 3, 4 μg; 4, 8 μg.
