# Supplementary material for: Inhibition of Host Vacuolar H+-ATPase Activity by a Legionella pneumophila Effector
Source: PLoS Pathog. 2010 Mar 19;6(3):e1000822. doi: 10.1371/journal.ppat.1000822 (PMC2841630; doi:10.1371/journal.ppat.1000822)
Supplement: Figure S4 — Interactions between v-ATPase and SidK deletion mutants in yeast. The indicated SidK deletionmutants (A) were expressed in yeast as GFP fusions. Total cell lysates were subjected to co-immunopreciitation with a GFP specific antibody and the presence of Vma1 and Vma2 in the precipitates were detected by immunoblot (B). The presence of Vma1 and Vma2 in the cell lyates was detected (C, upper panel) and the expression of the SidK truncations was evaluated with the GFP specific antibody (B, lower panel). (0.26 MB PDF) [file ppat.1000822.s008.pdf]

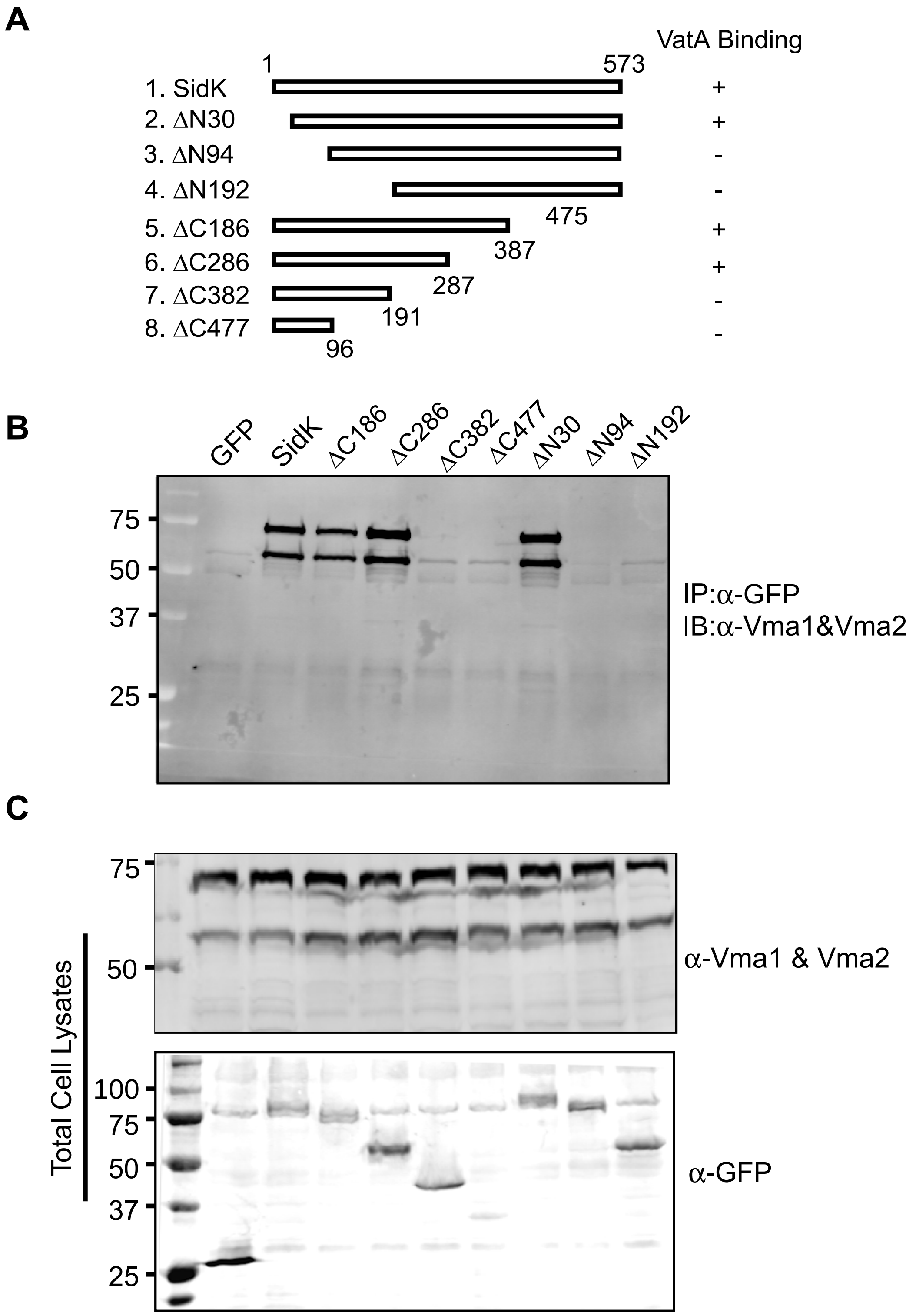

Fig. S4 Interactions between v-ATPase and SidK deletion mutants in yeast. The indicated SidK deletionmutants (A) were expressed in yeast as GFP fusions. Total cell lysates were subjected to co-immunopreciitation with a GFP specific antibody and the presence of Vma1 and Vma2 in the precipitates were detected by immunoblot (**B**). The presence of Vma1 and Vma2 in the cell lyates was detected (**C**, upper panel) and the expression of the SidK truncations was evaluated with the GFP specific antibody (**B**, lower panel).
