# Supplementary material for: Inhibition of Host Vacuolar H+-ATPase Activity by a Legionella pneumophila Effector
Source: PLoS Pathog. 2010 Mar 19;6(3):e1000822. doi: 10.1371/journal.ppat.1000822 (PMC2841630; doi:10.1371/journal.ppat.1000822)
Supplement: Figure S5 — Expression of sidK mutants in yeast. Indicated mutants were cloned into p425GPD, a start codon (ATG) was added to N-terminal deletion mutant. Samples were processed as described for Fig. 1 and proteins were detected with an anti-SidK antibody. (0.23 MB PDF) [file ppat.1000822.s009.pdf]

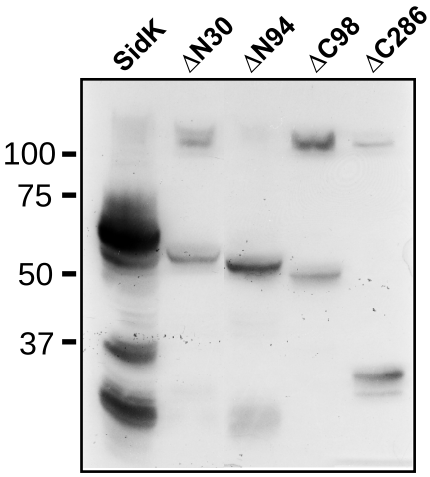

Fig. S5 Expression of sidK mutants in yeast. Indicated mutants were cloned into p425GPD, a start codon (ATG) was added to N-terminal deletion mutant. Samples were processed as described for Fig. 1 and proteins were detected with an anti-SidK antibody.
