# Supplementary material for: Inhibition of Host Vacuolar H+-ATPase Activity by a Legionella pneumophila Effector
Source: PLoS Pathog. 2010 Mar 19;6(3):e1000822. doi: 10.1371/journal.ppat.1000822 (PMC2841630; doi:10.1371/journal.ppat.1000822)
Supplement: Figure S6 — The SidK specific antibody did not affect SidK activity. v-ATPase assays were performed as described in Fig. 7A. 0.4 µM of SidK was used in each reaction. Indicated amount of antibody specific for SidJ or SidK was added 20 min after the addition of SidK. Reactions were allowed to proceed for 60 min and the release of free phosphate was measured. (0.08 MB PDF) [file ppat.1000822.s010.pdf]

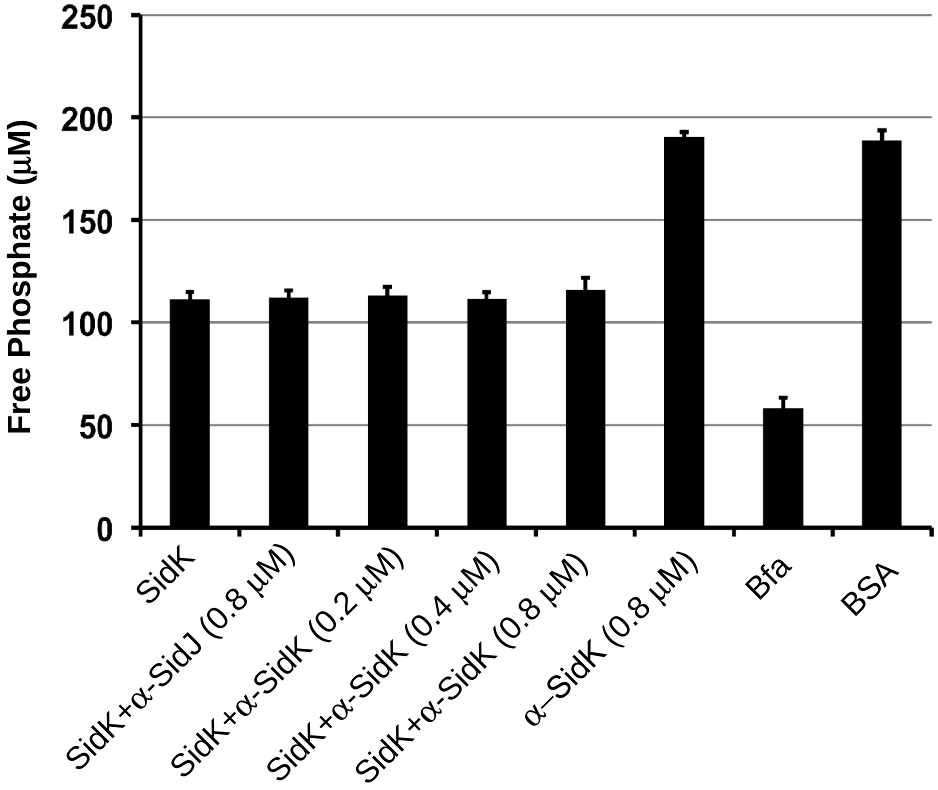


**Fig. S6** The SidK specific antibody did not affect SidK activity. v-ATPase assays were performed as described in Fig. 7A. 0.4 μM of SidK was used in each reaction. Indicated amount of antibody specific for SidJ or SidK was added 20 min after the addition of SidK. Reactions were allowed to proceed for 60 min and the release of free phosphate was measured.
