# Supplementary material for: Inhibition of Host Vacuolar H+-ATPase Activity by a Legionella pneumophila Effector
Source: PLoS Pathog. 2010 Mar 19;6(3):e1000822. doi: 10.1371/journal.ppat.1000822 (PMC2841630; doi:10.1371/journal.ppat.1000822)
Supplement: Figure S7 — SidK does not affect ATPase activity of Hsp70. Mammalian Hsp70 was purified from E. coli as a His6-tagged protein (A). 0.5 ug of purified protein was incubated with 2 mM ATP and the indicated compounds or proteins for various periods of time (X-axis). Hydrolysis of ATP was monitored by measuring released free phosphate with malachite green (B). Similar results were obtained in two experiments done in triplicates. Concentrations of testing materials: BSA, 1 µM; SidK, 1 µM; Vanadate, 1 mM; EDTA, 10 mM. (0.23 MB PDF) [file ppat.1000822.s011.pdf]

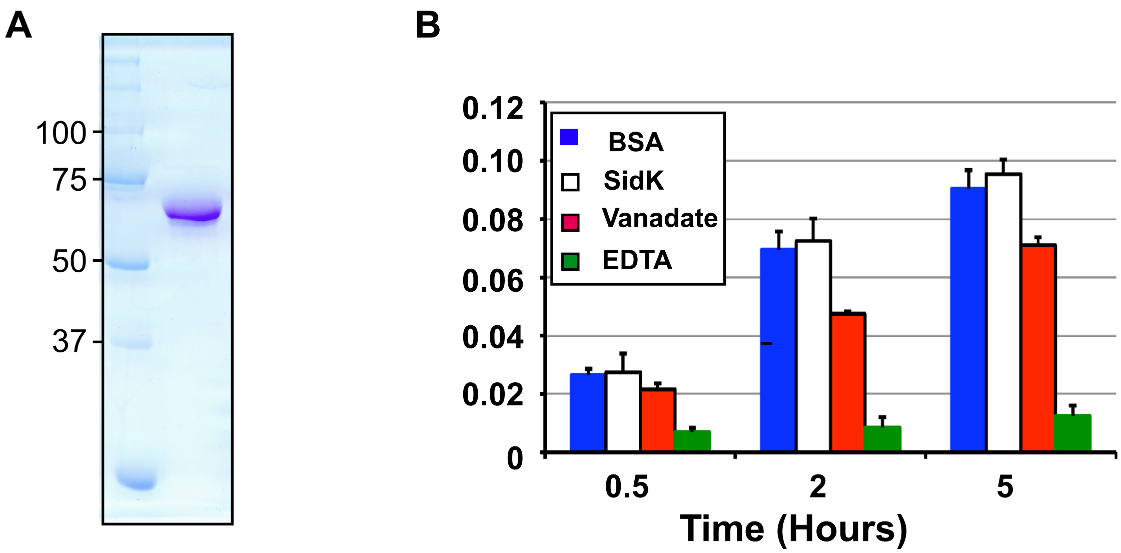

Fig. S7 SidK does not affect ATPase activity of Hsp70. Mammalian Hsp70 was purified from *E. coli* as a His_6-tagged protein (**A**). 0.5 ug of purified protein was incubated with 2 mM ATP and the indicated compounds or proteins for various periods of time (X-axis). Hydrolysis of ATP was monitored by measuring released free phosphate with malachite green (**B**). Similar results were obtained in two experiments done in triplicates. Concentrations of testing materials: BSA, 1 μM; SidK, 1 μM; Vanadate, 1 mM; EDTA, 10 mM.
